# Korea Hypertension Fact Sheet 2023: analysis of nationwide population-based data with a particular focus on hypertension in special populations

**DOI:** 10.1186/s40885-024-00262-z

**Published:** 2024-03-01

**Authors:** Hyeon Chang Kim, Hokyou Lee, Hyeok-Hee Lee, Dasom Son, Minsung Cho, Sojung Shin, Yeeun Seo, Eun-Jin kim, for the Korean Society of Hypertension (KSH)–Hypertension Epidemiology Research Working Group, Song Vogue Ahn, Sun Ha Jee, Sungha Park, Hae-Young Lee, Min Ho Shin, Sang-Hyun Ihm, Seung Won Lee, Jong Ku Park, Il Suh, Tae-Yong Lee

**Affiliations:** 1 Department of Preventive Medicine, Yonsei University College of Medicine, Seoul, Republic of Korea; 2 Institute for Innovation in Digital Healthcare, Yonsei University Health System, Seoul, Republic of Korea; 3 Department of Public Health, Yonsei University Graduate School, Seoul, Republic of Korea

**Keywords:** Hypertension, Prevalence, Awareness, Therapeutics, Korea

## Abstract

**Background:**

The Korea Hypertension Fact Sheet 2023, presented by the Korean Society of Hypertension, offers an overview of the prevalence and management of hypertension, along with recent trends.

**Methods:**

Data for the Fact Sheet were derived from the Korea National Health and Nutrition Examination Survey spanning 1998 to 2021, and the National Health Insurance Big Data from 2002 to 2021.

**Results:**

As of 2021, hypertension affected 28.0% of Korean adults aged 20 and older, totaling approximately 12.3 million individuals, with 5.3 million (43.5%) aged 65 or older. Among those with hypertension, awareness stood at 74.1%, treatment rates at 70.3%, and control rates at 56.0%. Over the years, the number of hypertension diagnoses increased from 3.0 million in 2002 to 11.1 million in 2021. During the same period, the utilization of antihypertensive medications rose from 2.5 million to 10.5 million, with treatment adherence also improving from 0.6 million to 7.8 million individuals. In 2021, the predominant antihypertensive drug class was angiotensin receptor blockers (75.1%), followed by calcium channel blockers (61.7%), diuretics (23.4%), and beta blockers (15.3%). Notably, 60.2% of all antihypertensive prescriptions involved combination therapy with at least two classes of antihypertensive medication. There was a positive trend towards stricter blood pressure control targets (systolic/diastolic blood pressure < 130/80 mmHg) among elderly hypertensive patients, as well as those with diabetes, obesity, and high-risk hypertension. However, this trend declined in individuals aged 80 years or older and those with chronic kidney disease in recent years.

**Conclusion:**

In Korea, hypertension management is making strides, yet the total number of hypertensive individuals is rising. Effectively addressing the growing population of elderly hypertensive patients and the persistently low treatment rates among younger individuals with hypertension is a critical challenge. Additionally, developing more efficient and customized policies for blood pressure control and cardiovascular disease prevention is imperative.

**Supplementary Information:**

The online version contains supplementary material available at 10.1186/s40885-024-00262-z.

## Background

Hypertension, characterized by elevated blood pressure levels, is a medical condition that significantly increases the risk of various diseases, including heart, brain, kidney, and others. Globally, it is a major contributor to premature mortality [1, 2]. Shockingly, the prevalence of hypertension has substantially increased, with estimates indicating a doubling from 648 million individuals in 1990 to a staggering 1,278 million in 2019. Alarmingly, only 38% of male hypertension patients and 47% of female hypertension patients are diagnosed and receiving treatment [3]. Despite declining age-adjusted cardiovascular disease mortality rates in Korea, heart disease and stroke are among the leading causes of death [4]. Furthermore, with the rapid aging of the population, we anticipate a surge in the absolute number of individuals with hypertension and cardiovascular diseases [5]. It is evident that effective blood pressure control is not only imperative for reducing the societal burden of disease but also for enhancing the quality of life on an individual level. Therefore, ongoing surveillance of hypertension prevalence and its management status is paramount. To address this, the Korean Society of Hypertension took the initiative by publishing its inaugural Hypertension Fact Sheet in 2018 and has been providing periodic updates since then [6–9].

In this study, we utilized nationally representative datasets to examine long-term trends in blood pressure distribution, prevalence of hypertension, hypertension management, and healthcare utilization for hypertension among the Korean population. Furthermore, in this issue, we have additionally assessed the management status of hypertension in special populations. This is particularly significant since there is still a scarcity of real-world data on the prevalence and management status of hypertension in these particular groups, despite modern hypertension treatment guidelines offering distinct treatment goals for them, such as elderly hypertensive patients or those with concurrent chronic conditions [10–13]. This approach enables us to establish a foundation for developing tailored treatment strategies for these unique groups and exploring improved approaches to hypertension management in the future.

## Methods

### Study populations

The Korea Hypertension Fact Sheet 2023 analyzed two nationally representative datasets. The first one is the Korea National Health and Nutrition Examination Survey (KNHANES) from 1998 to 2021. The KNHANES is a national surveillance system in Korea that assesses the health and nutritional status of the noninstitutionalized Korean population since 1998 [14, 15]. There have been 8 rounds of KNHANES between 1998 and 2018: KNHANES I (1998), KNHANES II (2001), KNHANES III (2005), KNHANES-IV (2007–2009), KNHANES V (2010–2012), KNHANES VI (2013–2015), KNHANES VII (2016–2018), and KNHANES VIII (2019–2021). The sample sizes and general characteristics of each round of KNHANES are presented in Table 1.

**Table 1:** General characteristics of participants in the Korea National Health and Nutrition Examination Survey (KNHANES), 1998–2021

**Characteristics**	**KNHANES by round**							
	**KNHANES I 1998 (*N* = 27,201)**	**KNHANES II 2001 (*N* = 26,854)**	**KNHANES III 2005 (*N* = 25,161)**	**KNHANES IV 2007–2009 (*N* = 18,210)**	**KNHANES V 2010–2012 (*N* = 19,394)**	**KNHANES VI 2013–2015 (*N* = 17,780)**	**KNHANES VII 2016–2018 (*N* = 19,197)**	**KNHANES VIII 2019–2021 (*N* = 18,511)**
**Response rate**	89.8%	77.3%	70.2%	74.5%	76.5%	74.1%	73.1%	70.7%
**Number (%)**								
Sex								
Male	12,853 (47.3)	12,647 (47.1)	11,722 (46.6)	7,836 (43.0)	8,378 (43.2)	7,709 (43.4)	8,465 (44.1)	8,270 (44.7)
Female	14,348 (52.7)	14,207 (52.9)	13,439 (53.4)	10,374 (57.0)	11,016 (56.8)	10,071 (56.6)	10,732 (55.9)	10,241 (55.3)
Age, year								
20–29	5,594 (20.6)	5,245 (19.5)	4,315 (17.1)	2,308 (12.7)	2,102 (10.8)	1,996 (11.2)	2,161 (11.3)	2,196 (11.9)
30–39	6,808 (25.0)	6,673 (24.8)	5,582 (22.2)	3,751 (20.6)	3,631 (18.7)	2,946 (16.6)	3,077 (16.0)	2,481 (13.4)
40–49	5,549 (20.4)	6,243 (23.2)	5,868 (23.3)	3,622 (19.9)	3,509 (18.1)	3,283 (18.5)	3,582 (18.7)	3,212 (17.4)
50–59	4,017 (14.8)	3,728 (13.9)	3,975 (15.8)	3,043 (16.7)	3,697 (19.1)	3,499 (19.7)	3,667 (19.1)	3,445 (18.6)
60–69	3,212 (11.8)	3,032 (11.3)	3,217 (12.8)	2,897 (15.9)	3,261 (16.8)	3,014 (17.0)	3,337 (17.4)	3,482 (18.8)
70 +	2,021 (7.4)	1,933 (7.2)	2,204 (8.8)	2,589 (14.2)	3,194 (16.5)	3,042 (17.1)	3,373 (17.6)	3,695 (20.0)
**Mean (95% CI)**								
Body mass index, kg/m^2^	23.2 (23.1–23.2)	23.5 (23.4–23.6)	23.6 (23.5–23.8)	23.6 (23.6–23.7)	23.7 (23.6–23.8)	23.8 (23.7–23.9)	24.0 (23.9–24.1)	24.1 (24.1–24.2)
Systolic blood pressure, mm Hg	125.0 (124.3–125.7)	122.8 (121.9–123.7)	118.0 (117.3–118.7)	115.5 (115.0–115.9)	117.6 (117.2–118.0)	116.9 (116.6–117.3)	117.8 (117.4–118.2)	118.7 (118.3–119.0)
Diastolic blood pressure, mm Hg	78.3 (77.9–78.8)	77.3 (76.8–77.9)	77.1 (76.5–77.7)	75.4 (75.1–75.7)	75.6 (75.3–75.9)	75.1 (74.8–75.3)	75.9 (75.7–76.1)	75.5 (75.2–75.7)
Fasting glucose, mg/dL	100.8 (99.5–102.0)	97.5 (96.7–98.3)	93.9 (93.2–94.6)	96.4 (95.9–96.8)	96.8 (96.3–97.2)	99.0 (98.5–99.4)	99.9 (99.5–100.4)	101.0 (100.5–101.4)
Total cholesterol, mg/dL	187.1 (185.8–188.4)	188.8 (187.7–190.0)	183.2 (181.9–184.5)	186.2 (185.4–187.0)	188.4 (187.6–189.2)	188.4 (187.7–189.2)	193.0 (192.3–193.8)	192.1 (191.3–192.8)
Triglyceride, mg/dL	121.4 (119.4–123.5)	138.8 (136.2–141.4)	133.9 (129.7–138.0)	135.0 (132.8–137.1)	134.6 (132.2–137.1)	139.3 (137.0–141.7)	139.5 (137.0–142.0)	132.9 (130.7–135.1)
LDL-cholesterol, mg/dL	113.3 (112.1–114.5)	114.8 (113.6–116.0)	113.1 (112.0–114.2)	110.7 (109.1–112.3)	113.6 (112.4–114.8)	114.5 (113.5–115.4)	118.9 (117.3–120.5)	116.8 (115.0–118.5)
HDL-cholesterol, mg/dL	50.0 (49.7–50.4)	46.2 (45.7–46.8)	45.0 (44.6–45.4)	48.1 (47.9–48.4)	49.7 (49.4–50.0)	51.0 (50.8–51.2)	51.0 (50.8–51.3)	52.2 (51.9–52.4)

*Abbreviations*: *KNHANES* Korea National Health and Nutrition Examination Survey, *LDL* low density lipoprotein, *HDL* high-density lipoprotein

The second is the National Health Insurance (NHI) Big Data from 2002 to 2021. Organized by the NHI Service, the NHI Big Data contains socio-demographics, hospital claims with International Classification of Diseases, 10th Revision (ICD-10, I10) coding, and mortality data of the entire population of the Republic of Korea [16]. Previously, the Korea Hypertension Fact Sheet 2018 analyzed adults aged 30 years from the KNHANES data and people of all ages in the NHI Big Data. Since the Korea Hypertension Fact Sheet 2020, both NHANES and NHI-Big Data were analyzed for adult data aged 20 or older [7].

### Analysis of the KNHANES from 1998 to 2021

Hypertension was defined as systolic blood pressure (SBP) ≥ 140 mmHg, diastolic blood pressure (DBP) ≥ 90 mmHg [11], or self-reported use of antihypertensive medication for the purpose of blood pressure control. Awareness rate was defined as the proportion of people with a physician diagnosis of hypertension among all people with hypertension. The treatment rate was defined as the proportion of people using antihypertensive drugs for 20 days or more per month among all people with hypertension. Control rate was defined as the proportion of people with SBP < 140 mmHg and DBP < 90 mmHg among (1) all people with hypertension and (2) people treated for hypertension. To evaluate the magnitude and management status of hypertension without the effects of population aging, age-standardized rates were calculated based on the demographics of the Korean population in 2005 according to the Population and Housing Census, Statistics Korea. To take into account the effect on estimator variance attributable to the KNHANES' stratified multistage clustered probability sampling design, we applied survey sampling weights to all the analyses.

The additional analysis for hypertension in special populations included individuals aged 65 and older, individuals aged 80 and older, individuals with diabetes, individuals with obesity, individuals with CKD, and individuals with hypertension and increased cardiovascular risk. Diabetes was defined as (1) having a fasting blood glucose level of 126 mg/dL or higher, (2) having received a medical diagnosis of diabetes, or (3) taking diabetes medication. Obesity was defined as having a body mass index (BMI) of 25 kg/m^2^ or higher, and CKD was defined as an estimated glomerular filtration rate (eGFR) less than 60 mL/min/1.73 m^2^, calculated using the CKD-EPI formula [17]. Hypertension and increased cardiovascular risk was defined as hypertension with three or more risk factors for cardiovascular disease, including old age (men ≥ 45 years, women ≥ 55 years; ≥ 65 years counted as two risk factors), family history of cardiovascular disease, smoking, obesity or abdominal obesity, dyslipidemia, pre-diabetes, or diabetes (counted as two risk factors) [13]. However, information on the use of dyslipidemia medications was missing in the 1998 and 2001 KNHANES surveys. Additionally, information on family history of cardiovascular disease was missing in the 1998, 2001, 2005, and 2007–2009 KNHANES surveys, and therefore was not included as a risk factor.

### Analysis of the NHI big data from 2002 to 2021

While the KNHANES data analysis defined hypertension based on measured blood pressure levels and the use of antihypertensive medication, the NHI Big Data analysis defined hypertension based on diagnosis codes, because the claim database did not have records of blood pressure measurements. Healthcare utilization was defined as at least one health insurance claim for diagnosis of essential hypertension (I10) each year. Treatment of hypertension was defined as at least one health insurance claim for hypertension diagnosis with an antihypertensive drug prescription each year. Adherence to treatment was defined as receiving prescriptions of antihypertensive drugs ≥ 290 days (80%) each year. Antihypertensive drugs were classified into diuretics (DU, thiazide-related and loop diuretics), beta-blockers (BB), calcium channel blockers (CCB), angiotensin-converting enzyme inhibitors (ACEi), angiotensin receptor blockers (ARB), potassium-sparing diuretics (PSD), or others (alpha-blockers, vasodilators, etc.). If the regimen of antihypertensive drugs had switched in a year, one with the longest duration was selected as the representative prescription of the patient for the given year.

The English version of the “Korea Hypertension Fact Sheet 2023” is attached as supplementary material for this manuscript. The Korean version is available at http://www.koreanhypertension.org/reference/guide.

## Results

### Trends of average blood pressure and hypertension prevalence

The average blood pressure of Korean adults has decreased between 1998 and 2008, but there has been little change in the last 13 years. Population mean SBP/DBP level was 119/74 mmHg for adults aged 20 years or older and 120/75 mmHg for those aged 30 years or older. Over the last 20 years, the age-standardized mean blood pressure levels have decreased yet without significant change in recent years. The age-standardized prevalence of hypertension among adults aged 20 years or older also modestly decreased from 26.0% (men 29.6%, women 22.3%) in 1998 to 21.8% (men 25.7%, women 17.4%) in 2021. Over the same period, the age-standardized prevalence of hypertension among adults aged 30 years or older decreased from 30.7% (men 33.4%, women 27.4%) to 26.8% (men 31.5%, women 21.7%) (Table 2; Supplement, page 7–9).

**Table 2:** Crude and age-standardized prevalence of hypertension, 1998–2021

**Year**	**Crude prevalence of hypertension, %**						**Age-standardized prevalence of hypertension, %**^**a**^					
	**Adults aged 20 years or older**			**Adults aged 30 years or older**			**Adults aged 20 years or older**			**Adults aged 30 years or older**		
	**Total**	**Male**	**Female**	**Total**	**Male**	**Female**	**Total**	**Male**	**Female**	**Total**	**Male**	**Female**
1998	25.1	28.5	22.1	29.7	32.0	27.6	26.0	29.6	22.3	30.7	33.4	27.4
2001	26.0	30.7	22.7	29.8	34.4	26.6	23.6	28.4	20.2	28.5	33.1	25.3
2005	22.9	25.5	20.4	28.1	30.9	25.4	22.7	26.1	18.9	28.0	31.5	23.8
2007	20.4	21.5	19.3	25.0	26.4	23.7	19.7	21.7	17.3	24.4	26.7	21.5
2008	22.7	23.5	21.9	27.6	28.5	26.6	21.4	23.2	19.1	26.5	28.5	24.0
2009	23.4	25.9	20.9	28.2	31.2	25.3	21.4	25.2	17.4	26.5	30.8	21.9
2010	24.1	25.3	23.0	28.9	30.1	27.7	21.7	24.1	18.8	26.8	29.3	23.8
2011	26.5	29.8	23.3	30.8	33.9	27.8	23.9	28.6	18.8	28.4	32.8	23.6
2012	26.7	28.4	25.1	31.5	33.3	29.8	23.8	26.9	20.3	28.9	32.1	25.2
2013	25.7	28.9	22.8	30.4	34.2	26.9	22.4	26.9	17.7	27.2	32.4	22.1
2014	24.3	26.4	22.2	28.9	31.8	26.2	20.5	24.1	16.6	25.4	29.7	20.9
2015	27.0	29.5	24.5	32.0	35.1	29.1	22.5	26.7	18.2	27.8	32.6	22.9
2016	28.5	31.9	25.1	33.5	37.7	29.4	23.7	28.6	18.4	29.1	35.0	22.9
2017	26.9	30.4	23.5	31.2	35.0	27.6	22.3	27.4	16.9	26.9	32.3	21.3
2018	28.8	31.5	26.0	33.3	36.4	30.4	23.5	28.0	18.6	28.3	33.2	23.1
2019	28.4	29.7	27.0	32.9	34.7	31.2	22.5	25.9	18.8	27.2	31.1	22.8
2020	29.4	33.5	25.4	34.2	38.9	29.7	23.3	29.1	17.0	28.3	34.9	21.3
2021	28.4	30.4	26.4	33.2	35.8	30.7	21.8	25.7	17.4	26.8	31.5	21.7

^a^Age-standardized prevalence was calculated using the 2005 population projections for Korea

Before the age of 60, the prevalence of hypertension is higher in men than in women. However, in the age 60 s, the prevalence is similar between men and women, and after the age of 70, the prevalence of hypertension becomes higher in women. The absolute number of people with hypertension has steadily increased along with the rapid aging of the population; 12.3 million Korean adults have hypertension as of 2021. In particular, the number of elderly women with hypertension has increased rapidly. In 2021, estimated people with hypertension were 4.3 million men and 2.6 million women under the age of 65, but 2.2 million men and 3.2 million women aged 65 years or older (Supplement, page 10–11).

### Trends of hypertension management

Over the past two decades, there has been significant improvement in the management of hypertension, including awareness, treatment, and control rates. In 2021, among adults aged 20 and older with hypertension, the awareness rate was 74.1%, indicating that they were aware of their condition. The treatment rate was 70.3%, representing the proportion receiving treatment, and the control rate was 56.0%, meaning those who had their blood pressure controlled below 140/90 mmHg. These figures showed an improvement compared to the previous year's data, where the awareness rate was 69.5%, the treatment rate was 64.8%, and the control rate was 47.4%. However, the degree of management varied greatly by age and sex. All management indices tended to be higher in older adults than in younger adults, and higher in women than in men. However, gender-difference varies depending on age. Women under the age of 50 have higher awareness, treatment, and control rates compared to men of same age. After the age of 60, the awareness and treatment rates become similar in men and women, and the control rate is even lower in women than in men (Supplement, pages 13–16).

The average blood pressure levels in individuals with hypertension have shown a consistent decline, albeit with varying degrees of change across different age groups. Among those aged 65 and older, the average SBP dropped significantly from 159.5 mmHg in 1998 to 134.7 mmHg in the period of 2019–2021. Conversely, in the 40–64 age group, it decreased from 148.4 to 131.6 during the same timeframe, while there was minimal change observed in hypertensive individuals aged 20–39. A similar pattern was observed for DBP, with a decrease in older hypertensive individuals and relative stability in the younger age group. When limited to individuals receiving treatment for hypertension, SBP and DBP decreased across all age groups, albeit with varying degrees of reduction. This implies that the primary reason for the lack of blood pressure reduction in younger hypertensive individuals is their poor-compliance with hypertension treatment (Table 3; Supplement, pages 17–18).

**Table 3:** The average blood pressure levels among people with hypertension and people treated for hypertension, 1998–2021

**Year**	**Average systolic blood pressure, mmHg**						**Average diastolic blood pressure, mmHg**					
	**People with hypertension**			**People treated for hypertension**			**People with hypertension**			**People treated for hypertension**		
	**Age 20–39**	**Age 40–64**	**Age 65 + **	**Age 20–39**	**Age 40–64**	**Age 65 + **	**Age 20–39**	**Age 40–64**	**Age 65 + **	**Age 20–39**	**Age 40–64**	**Age 65 + **
1998	139.6	148.4	159.5	139.1	148.0	160.4	93.8	92.7	85.7	91.2	91.3	85.8
2001	137.6	144.9	151.4	148.1	142.9	146.6	93.2	90.8	84.5	92.8	88.6	82.3
2005	130.7	137.5	143.7	125.2	132.9	138.7	93.7	90.2	82.1	82.4	85.4	79.6
2007–2009	132.7	133.9	137.3	128.3	128.6	132.6	94.0	87.7	78.3	87.0	82.7	76.4
2010–2012	133.5	135.1	136.1	125.4	128.6	132.8	94.0	86.9	75.1	85.5	81.2	73.6
2013–2015	134.6	132.8	134.0	125.6	126.3	130.6	94.4	85.9	73.0	84.4	80.8	71.4
2016–2018	132.9	132.2	134.0	121.4	125.7	130.9	93.3	85.9	73.0	82.2	80.6	71.4
2019–2021	135.5	131.6	134.7	125.7	125.0	131.6	92.7	85.1	74.1	82.0	80.2	72.7

### Healthcare utilization for hypertension

Healthcare utilization patterns related to hypertension have undergone significant changes over the past two decades. The number of individuals diagnosed with hypertension has surged from 3.0 million in 2002 to 11.8 million in 2021, marking a 3.7-fold increase. Likewise, the count of people using antihypertensive medications has risen from 2.5 million in 2002 to 10.5 million in 2021, reflecting a 4.1-fold increase. Notably, the number of individuals demonstrating good adherence to antihypertensive medication has shown remarkable growth, escalating from 0.6 million in 2002 to 7.8 million in 2021. Among the 10.5 million individuals undergoing treatment for hypertension in 2021, 61.2% were concurrently receiving treatment for dyslipidemia, 27.7% were receiving diabetes treatment, and 22.2% were undergoing treatment for both dyslipidemia and diabetes (Supplement, page 20–21).

The adoption of combination therapy has rapidly expanded, with 39.8% utilizing one class, 43.8% employing two classes, and 16.4% using three or more classes of antihypertensive drugs in 2021 (Table 4; Supplement, page 22). In 2021, the most frequently prescribed antihypertensive drug class was ARB (75.1%), followed by CCB (61.7%), DU (23.4%), BB (15.3%), PSD (1.8%), and ACEi (1.3%). The most commonly prescribed regimen for hypertension treatment was dual therapy involving ACEi/ARB plus CCB, followed by ARB monotherapy and CCB monotherapy (Supplement, page 24–25). It is noteworthy that patients who were treating diabetes or dyslipidemia in conjunction with hypertension tended to use combination therapy more frequently than those solely treating hypertension. Moreover, among male patients, the usage frequency of ACEi/ARB was higher compared to female patients, while the usage frequency of DU was lower (Supplement, page 26–27).

**Table 4:** Trends of antihypertensive medication use,2002–2021

**Year**	**Treated total**	**Monotherapy**		**Dual therapy**		**3 classes or more**	
	**Number (×1,000)**	**Number (×1,000)**	**Percent**	**Number (×1,000)**	**Percent**	**Number (×1,000)**	**Percent**
2002	2,523	1,434	56.9	785	31.1	303	12.0
2003	3,213	1,669	52.0	1,088	33.9	456	14.2
2004	3,720	1,794	48.2	1,321	35.5	606	16.3
2005	4,468	2,045	45.8	1,624	36.3	799	17.9
2006	4,993	2,201	44.1	1,831	36.7	961	19.2
2007	5,398	2,338	43.3	1,973	36.6	1,087	20.1
2008	5,770	2,454	42.5	2,121	36.8	1,194	20.7
2009	6,182	2,536	41.0	2,352	38.0	1,294	20.9
2010	6,538	2,578	39.4	2,555	39.1	1,405	21.5
2011	6,772	2,627	38.8	2,721	40.2	1,424	21.0
2012	7,220	2,815	39.0	2,931	40.6	1,474	20.4
2013	7,499	2,965	39.5	3,078	41.0	1,456	19.4
2014	7,696	3,111	40.4	3,211	41.7	1,374	17.9
2015	7,944	3,251	40.9	3,334	42.0	1,359	17.1
2016	8,297	3,409	41.1	3,516	42.4	1,372	16.5
2017	8,633	3,534	40.9	3,710	43.0	1,389	16.1
2018	9,027	3,672	40.7	3,905	43.3	1,450	16.1
2019	9,512	3,865	40.6	4,127	43.4	1,520	16.0
2020	9,914	3,975	40.1	4,327	43.6	1,612	16.3
2021	10,455	4,161	39.8	4,579	43.8	1,714	16.4

### Hypertension in special populations

Among hypertensive individuals aged 65 and older, 59.4% had SBP < 140 mmHg and DBP < 90 mmHg. Among those aged 80 and older, 55.7% had SBP/DBP readings below 140/90 mmHg. The same figures for hypertensive individuals with diabetes were 63.0%, for those with obesity were 50.8%, for those with CKD were 58.2%, and for those with high-risk hypertension were 52.4% (Supplement, page 29).

Among hypertensive individuals aged 65 and older, the proportion with SBP/DBP < 130/80 mmHg was only 1.7% in 1998, but it steadily increased to 37.6% in the period of 2016–2019 and recorded 36.0% in 2019–2021. Among hypertensive individuals aged 80 and older, the same proportion was 2.2% in 1998, and it climbed to 38.6% in 2016–2019, then decreased to 35.8% in 2019–2021. Among hypertensive patients with diabetes, the proportion with SBP/DBP < 130/80 mmHg increased from 5.2% in 1998 to 37.3% in 2019–2021. In hypertensive patients with obesity, the same measure increased from 2.1% to 28.3%. Among hypertensive patients at high risk for cardiovascular disease, this proportion increased from 2.4% to 28.6%. Conversely, among hypertensive patients with CKD, the same measure rose from 4.7% in 1998 to 43.0% in 2013–2015 but decreased to 34.5% in 2019–2021 (Table 5; Supplement, page 30–41).

**Table 5:** Changes in hypertension control rates among special populations, 1998–2001

**Year**	**Aged 65 years or older**		**With diabetes**		**With obesity**^a^		**With chronic kidney disease**^b^		**At high cardiovascular risk**^c^	
	**Proportion of SBP/DBP 130–139/80–89 mmHg, %**	**Proportion of SBP/DB*P* < 130/80 mmHg, %**	**Proportion of SBP/DBP 130–139/80–89 mmHg, %**	**Proportion of SBP/DB*P* < 130/80 mmHg, %**	**Proportion of SBP/DBP 130–139/80–89 mmHg, %**	**Proportion of SBP/DB*P* < 130/80 mmHg, %**	**Proportion of SBP/DBP 130–139/80–89 mmHg, %**	**Proportion of SBP/DB*P* < 130/80 mmHg, %**	**Proportion of SBP/DBP 130–139/80–89 mmHg, %**	**Proportion of SBP/DB*P* < 130/80 mmHg, %**
1998	4.9	1.7	6.1	5.2	5.7	2.1	5.7	4.7	5.5	2.4
2001	11.3	5.3	7.5	7.3	10.3	4.6	13.3	4.8	9.0	4.0
2005	17.1	16.4	20.4	17.3	17.3	8.2	20.3	18.8	18.3	10.8
2007–2009	22.6	28.6	25.5	27.2	22.2	19.0	26.1	29.4	22.8	23.5
2010–2012	22.4	33.2	26.4	29.4	21.4	20.6	23.6	39.1	20.8	22.9
2013–2015	22.6	37.4	20.8	36.1	21.5	23.8	20.0	43.0	20.3	25.6
2016–2018	22.8	37.6	23.9	35.8	21.7	25.4	24.1	39.6	22.7	27.7
2019–2021	23.4	36.0	25.7	37.3	22.5	28.3	23.7	34.5	23.7	28.6

*Abbreviations*: *SBP* systolic blood pressure, *DBP* diastolic blood pressure

^a^Body mass index ≥ 25 kg/m^2^

^b^Estimated glomerular filtration rate (eGFR) < 60 mL/min/1.73 m^2^

^c^Having ≥ 3 risk factors, including old age, family history of cardiovascular disease, smoking, obesity, dyslipidemia, diabetes (counted as two), or prediabetes

## Discussion

The Korea Hypertension Fact Sheet 2023 offers an extensive review of the scale and management status of hypertension in Korea over the past two decades. While the average blood pressure and hypertension prevalence in the population have shown relative stability in recent years, the absolute number of individuals living with hypertension has steadily risen, surpassing 12 million due to the aging population. Particularly concerning is the swift increase in the number of elderly individuals with hypertension, especially among elderly women. This trend could lead to a significant escalation in the burden of hypertension and its associated complications. Fortunately, there have been notable improvements in awareness, treatment, and treatment adherence rates within this older age group. However, these rates remain relatively low among younger individuals with hypertension. Consequently, while average blood pressure levels are decreasing among the elderly, there is not a significant decrease in younger age groups. To address this, it is imperative to allocate more resources toward blood pressure control and cardiovascular disease prevention, especially for the growing elderly hypertensive population. Additionally, dedicated efforts are needed to enhance the detection and treatment of hypertension among younger age groups.

Although the number of people with hypertension is increasing, it is a positive development that more of them are being diagnosed and prescribed antihypertensive medications, especially those who are consistently receiving treatment. However, to mitigate the burden of hypertension-related diseases, it is crucial to enhance not only the diagnosis and treatment of hypertension but also primary prevention measures. With the aging population and a rapid surge in obesity among younger age groups, concerns about the burden of hypertension-related diseases are escalating across all age ranges. The growing number of hypertensive patients with concomitant chronic conditions such as diabetes and dyslipidemia exacerbate these concerns. While Korea is considered as a global leader in improving hypertension treatment and reducing cardiovascular disease mortality rates [3, 4, 18], failing to address these issues may hinder our ability to effectively manage the societal burden posed by hypertension and its complications.

Similar to many other guidelines, the Korean Society of Hypertension's hypertension treatment guidelines also provide tailored hypertension treatment strategies and target blood pressure levels for various special subgroups, in addition to general treatment plans [13, 19]. However, there has been a lack of Korean data on blood pressure management levels for different subgroups. In this edition of the Hypertension Fact Sheet, we have utilized data from the KNHANES to illustrate changes in blood pressure levels among select groups that can be identified, including the elderly, individuals with diabetes, individuals with obesity, individuals with CKD, and those at high risk of cardiovascular disease. Among elderly hypertensive patients, we have observed a significant increase in the proportion of those with SBP/DBP < 130/80 mmHg and SBP/DBP 130–139/80–89 mmHg. However, it has recently been noted that the proportion of individuals with SBP/DBP < 130/80 mmHg is beginning to decline. Further investigation is needed to determine whether this change is in response to recent hypertension treatment guidelines recommending against overly aggressive blood pressure reduction in the elderly or if other factors are at play. Guidelines recommend a more aggressive approach to blood pressure control in cases where other chronic conditions such as diabetes or CKD coexist, especially when there is suspicion of target organ damage [13, 20, 21]. While there has been an increase in the proportion of hypertensive patients with diabetes or those at high cardiovascular risk achieving SBP/DBP < 130/80 mmHg, these rates remain relatively low. Among hypertensive patients with CKD, the proportion of those achieving SBP/DBP < 130/80 mmHg has increased but started declining since 2016. Adequate blood pressure management in CKD patients is critical for preventing cardiovascular diseases and mortality [22, 23]. However, further research is needed to determine the appropriate blood pressure management for CKD patients, as shifts in the proportion of CKD patients with accompanying complications or changes in the elderly population also influence blood pressure distributions.

The most significant aspect of the Korea Hypertension Fact Sheet is its applicability. The KNHANES provides an unbiased sample of the Korean population, and the NHI Big Data covers medical service utilization for the entire Korean population. However, there are several limitations that should be acknowledged. First, the KNHANES is based on non-institutionalized Korean residents, which may exclude individuals with severe illnesses. Second, using the I10 code as the operational definition for hypertension was done to maintain consistency across successive issues of the Hypertension Fact Sheet. However, it is recognized that this approach may not be ideal for identifying individuals with hypertension, especially among older patients with hypertensive complications or organ damage. Third, there were variations in data collection methods and survey details within the KNHANES despite standardized protocols and rigorous quality control measures. These variations might have affected the analysis of trends over time. Fourth, the NHI Big Data may not be the most suitable source for identifying disease occurrence and prevalence since the data primarily serve medical service claims and reimbursement purposes. Fifth, adherence to antihypertensive medication was assessed based on prescriptions, potentially leading to an overestimation as it cannot confirm whether the medication was actually taken. Lastly, the identification of special populations relied on available examination results from the KNHANES data, leading to a lack of information on the duration of diabetes and CKD, as well as complications. This limitation prevented a more detailed risk assessment. Additionally, the definition of the high cardiovascular risk group does not align with Korean Society of Hypertension guidelines.

## Conclusions

While the average blood pressure and the prevalence of hypertension have remained relatively stable in the Korean population, the rapid aging of the demographic and increased lifespan will inevitably lead to a continuous rise in the absolute burden of hypertension and its associated complications. Despite recent advancements in hypertension management, proactive blood pressure control is still essential, especially for young adults aged 20–39 and those at greater risk of cardiovascular disease. It's also crucial to avoid overly aggressive blood pressure reduction in vulnerable populations. In addition to preventing complications and fatalities linked to hypertension, it is of paramount importance to emphasize the significance of treatment adherence and the maintenance of optimal blood pressure levels as top priorities. Furthermore, ongoing monitoring of blood pressure distributions within special populations is vital. This facilitates the development of customized blood pressure control strategies tailored to specific subgroups. Hence, the imperative task ahead is to formulate tailored prevention and management strategies that are comprehensive and well-suited for diverse subpopulations.

### Supplementary Information

**Additional file 1. **

## Data Availability

The datasets used and/or analyzed during the current study are available from the corresponding author on reasonable request.

## Abbreviations

ACEi: Angiotensin-converting enzyme inhibitor
ARB: Angiotensin receptor blockers
BB: Beta-blockers
CCB: Calcium channel blockers
CKD: Chronic kidney disease
DBP: Diastolic blood pressure
DU: Diuretics
ICD-10: International Classification of Diseases, 10th Revision
KNHANES: Korea National Health and Nutrition Examination Survey
NHI: National Health Insurance
PSD: Potassium-sparing diuretic
SBP: Systolic blood pressure
