# Supplementary material for: Korea Hypertension Fact Sheet 2023: analysis of nationwide population-based data with a particular focus on hypertension in special populations
Source: Clin Hypertens. 2024 Mar 1;30:7. doi: 10.1186/s40885-024-00262-z (PMC10905929; doi:10.1186/s40885-024-00262-z)
Supplement: Supplementary file 1 — Additional file 1. [file 40885_2024_262_MOESM1_ESM.pdf]

KOREA HYPERTENSION FACT SHEET 2023

The Korean Society of Hypertension

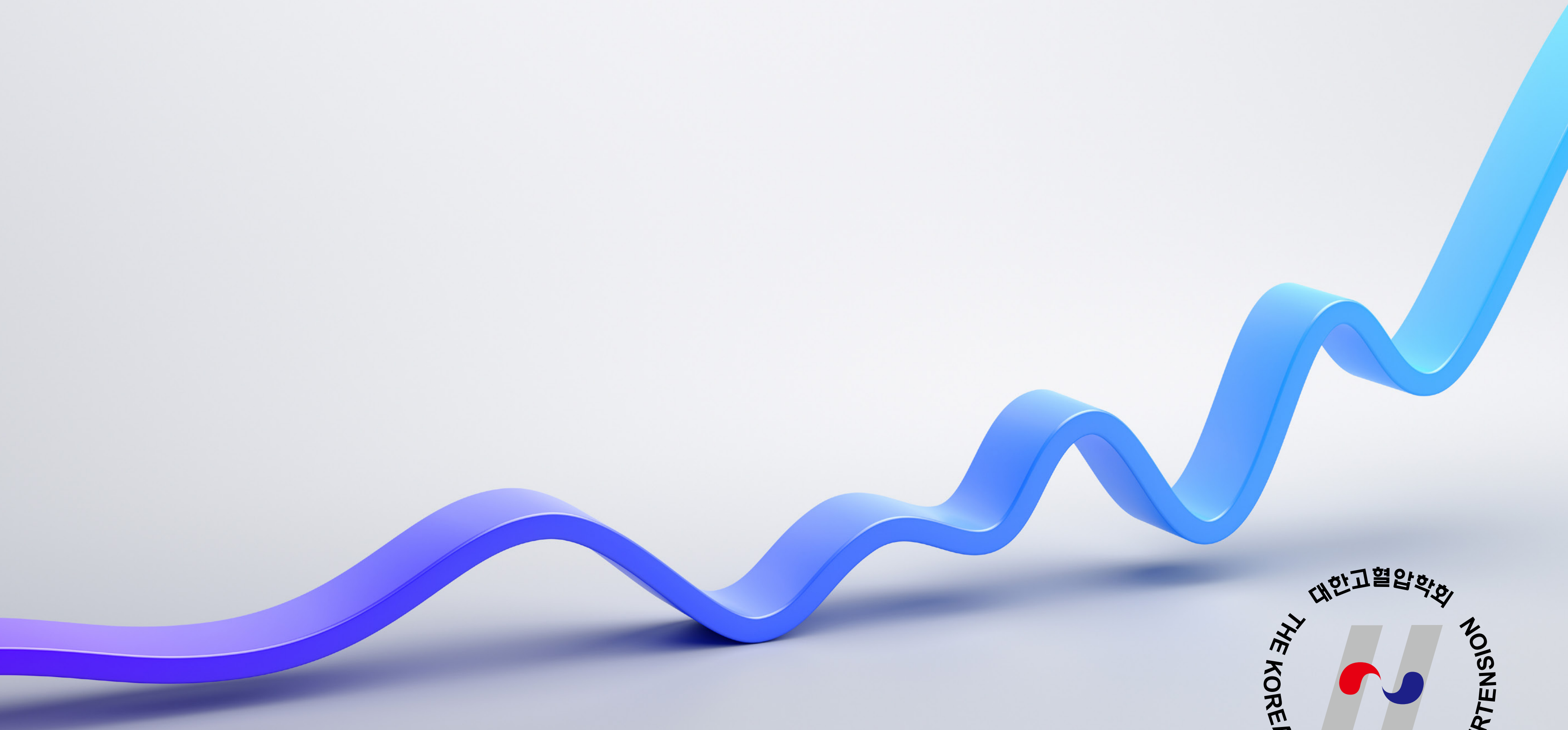

대한고혈압학회
THE KOREAN SOCIETY OF HYPERTENSION

Methods

Korea National Health and Nutrition Examination Survey

Study Population		Adults aged 20 years or older / 1998 to 2021
Definition of Hypertension		① Systolic blood pressure (SBP) ≥140 mmHg, or<br>② Diastolic blood pressure (DBP) ≥90 mmHg, or<br>③ Taking antihypertensive medication
Hypertension Management	Awareness rate	Proportion of people with physician diagnosis of hypertension among those with hypertension
	Treatment rate	Proportion of people taking antihypertensive medication for ≥20 days/month among those with hypertension
	Control rate (among prevalent)	Proportion of people with SBP <140 mmHg and DBP <90 mmHg among those with hypertension
	Control rate (among treated)	Proportion of people with SBP <140 mmHg and DBP <90 mmHg among those taking antihypertensive medication

Korea National Health Insurance Big Data

Study Population		Adults aged 20 years or older / 2002 to 2021
Healthcare Utilization	Diagnosis	≥1 health insurance claim for hypertension diagnosis (ICD-10: I10) each year
	Treatment	≥1 health insurance claim for hypertension diagnosis and antihypertensive prescription each year
	Adherence	Antihypertensive prescription ≥290 days (80%) each year
Antihypertensive Treatment	Regimen	The combination of antihypertensive classes in a prescription; if the regimen is switched, one with the longest duration is selected for a given year
	Antihypertensive class	Diuretics (DU; thiazide and related diuretics, loop diuretics), Angiotensin converting enzyme inhibitors (ACEi), Angiotensin receptor blockers (ARB), Calcium channel blockers (CCB), Beta-blockers (BB), Potassium-sparing diuretics (PSD), Others (ETC; alpha-blockers, vasodilators, etc.)

Contents

Trends of Average Blood Pressure and Hypertension Prevalence

7 Trends of Average Blood Pressure
8 Trends of Hypertension Prevalence
10 Trends of Hypertension Prevalence by Sex and Age
11 Trends of Number of People with Hypertension

Trends of Hypertension Management

13 Trends of Awareness Rate by Sex and Age
14 Trends of Treatment Rate by Sex and Age
15 Trends of Control Rate by Sex and Age
17 Trends of Average Blood Pressure

Healthcare Utilization for Hypertension

20 Trends of Healthcare Utilization for Hypertension
21 Trends of Co-treatment for Dyslipidemia and Diabetes
22 Trends of Antihypertensive Medication Use
24 Composition of Antihypertensive Treatment
26 Antihypertensive Medication Use By Co-treatment Status

Hypertension in Special Populations

29 Trends of Blood Pressure Control in Special Populations
30 Trends of Blood Pressure Control in the Elderly (Age 65+)
32 Trends of Blood Pressure Control in the Elderly (Age 80+)
34 Trends of Blood Pressure Control in People with Diabetes
36 Trends of Blood Pressure Control in People with Obesity
38 Trends of Blood Pressure Control in People with Chronic Kidney Disease
40 Trends of Blood Pressure Control in People with High-risk Hypertension
41 Trends of Blood Pressure Control in People Treated for High-risk Hypertension

THE KOREAN SOCIETY OF HYPERTENSION 대한고혈압학회

KOREA HYPERTENSION FACT SHEET 2023

Summary of Hypertension Statistics (Age 20+)

12.3 million
have hypertension

11.1 million
are diagnosed

10.5 million
are treated

7.8 million
are adherent to treatment

Data Source: Korea National Health and Nutrition Examination Survey 2021, Korea National Health Insurance Big Data 2021

Among age 20+

Hypertension Prevalence

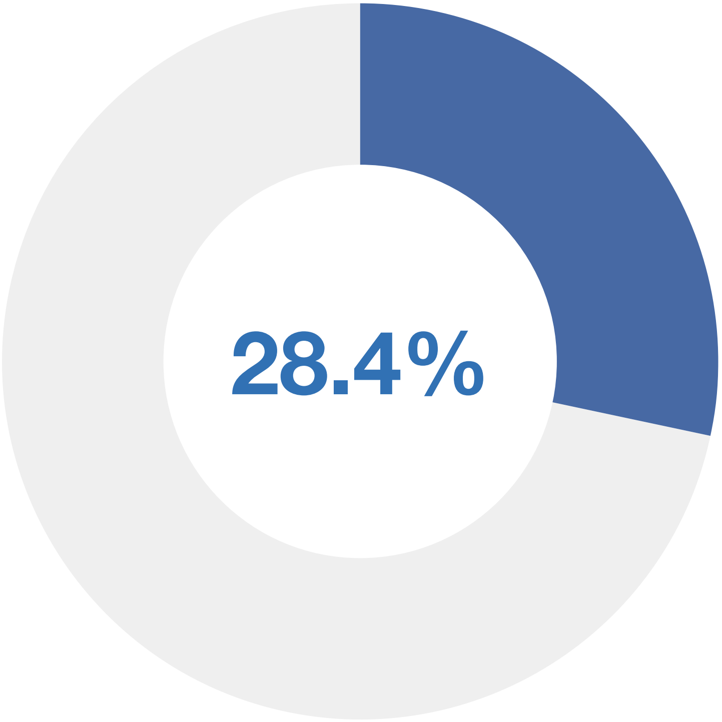

Among age 20+ with hypertension

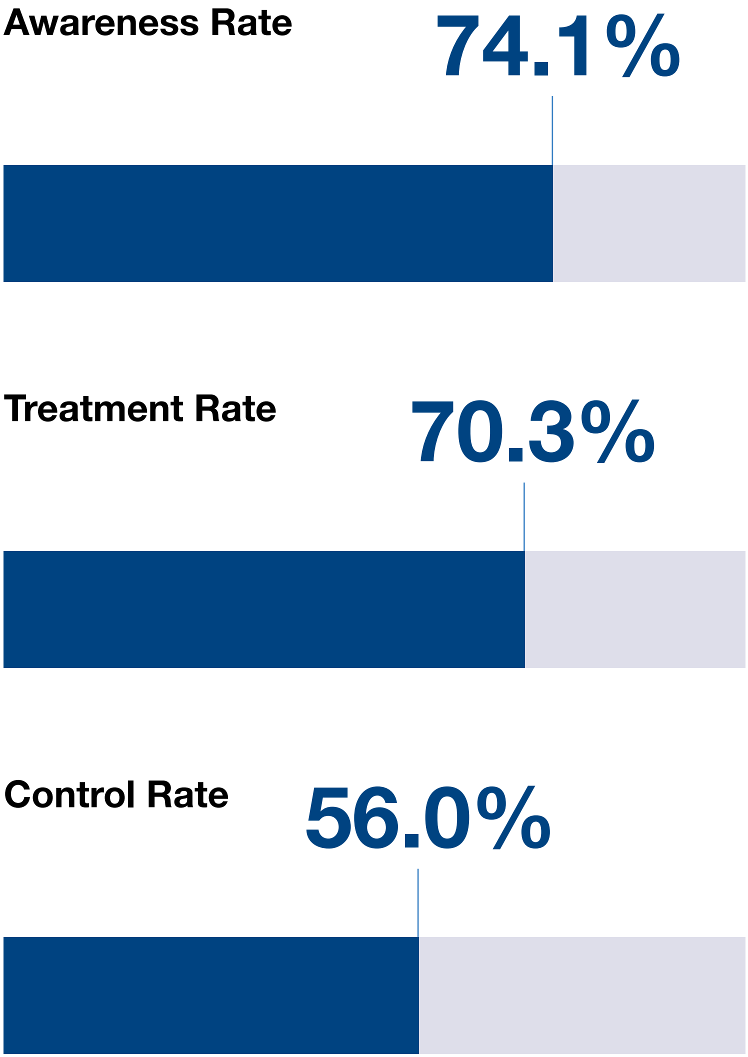

Data Source: Korea National Health and Nutrition Examination Survey 2021

Trends of Average Blood Pressure and Hypertension Prevalence

Trends of Average Blood Pressure
Trends of Hypertension Prevalence
Trends of Hypertension Prevalence by Sex and Age
Trends of Number of People with Hypertension

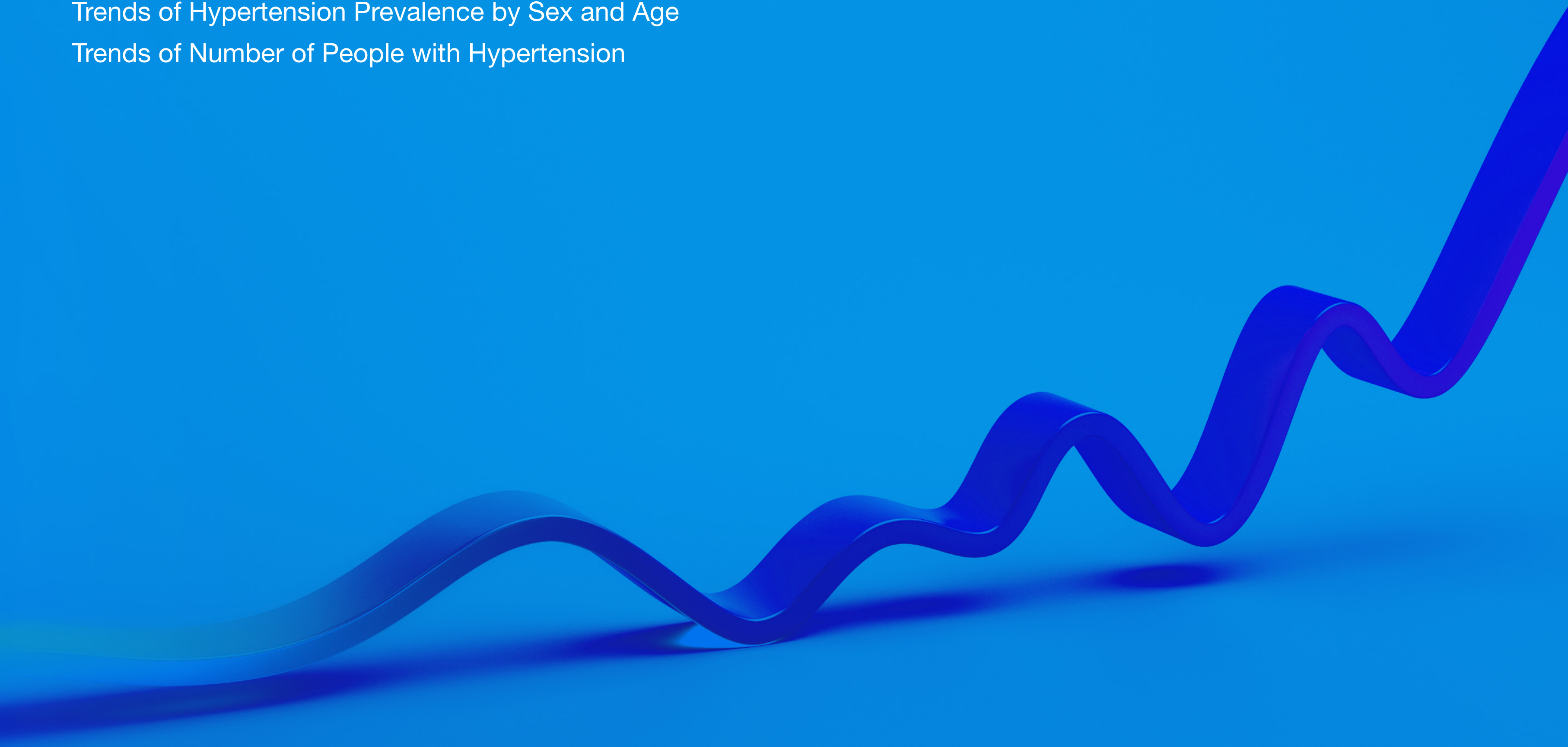

Trends of Average Blood Pressure

(Age 20+, Age-standardized)

	Crude	Age-standardized
Age 20+	**119/74** mmHg	**117/74** mmHg
Age 30+	**120/75** mmHg	**118/75** mmHg

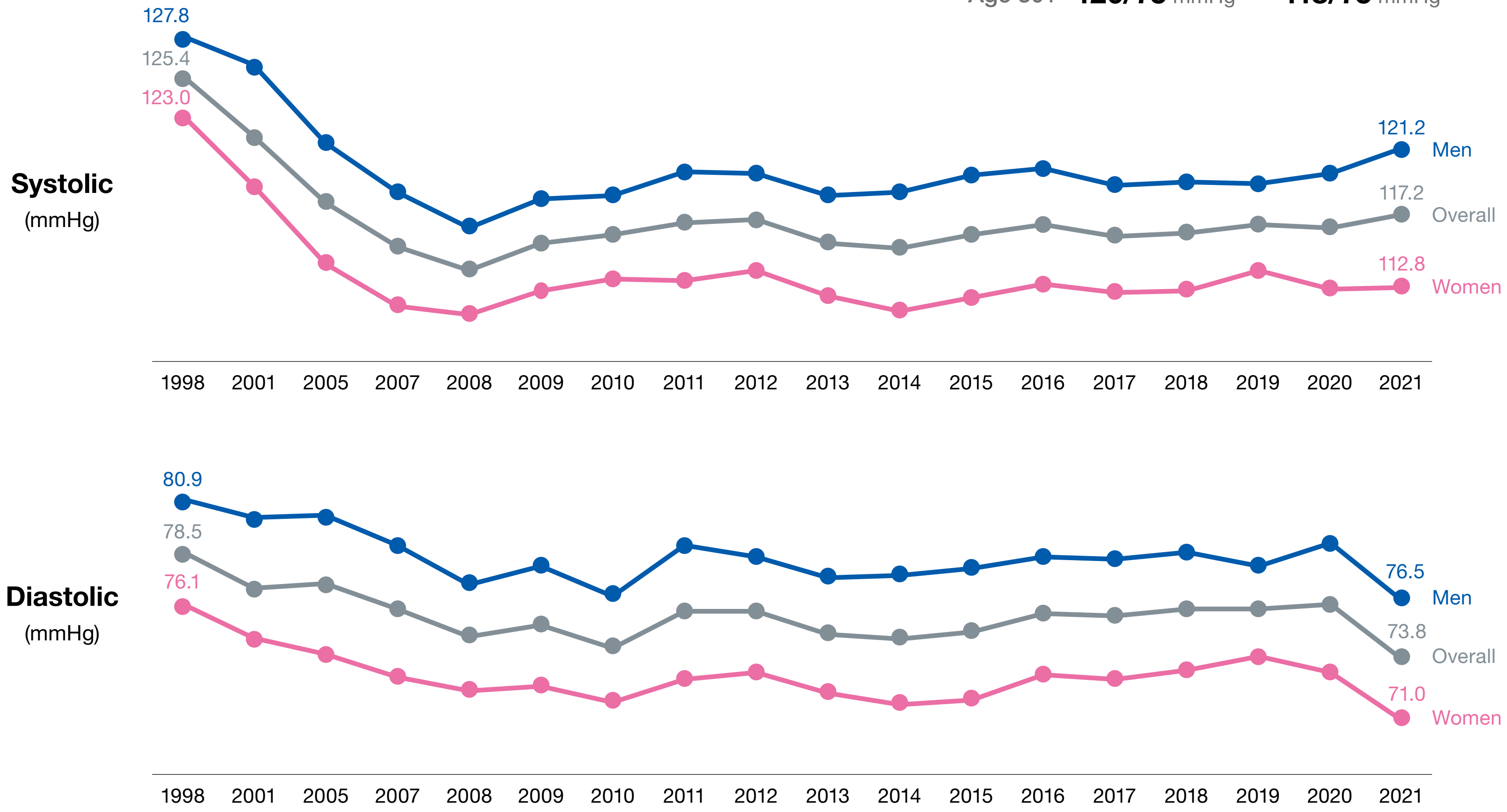

Data Source: Korea National Health and Nutrition Examination Survey 1998-2021 (directly age-standardized to the 2005 projected population)

Trends of Hypertension Prevalence

(Age 20+, Age-standardized)

	Crude	Age-standardized
Age 20+	**28** %	**22** %

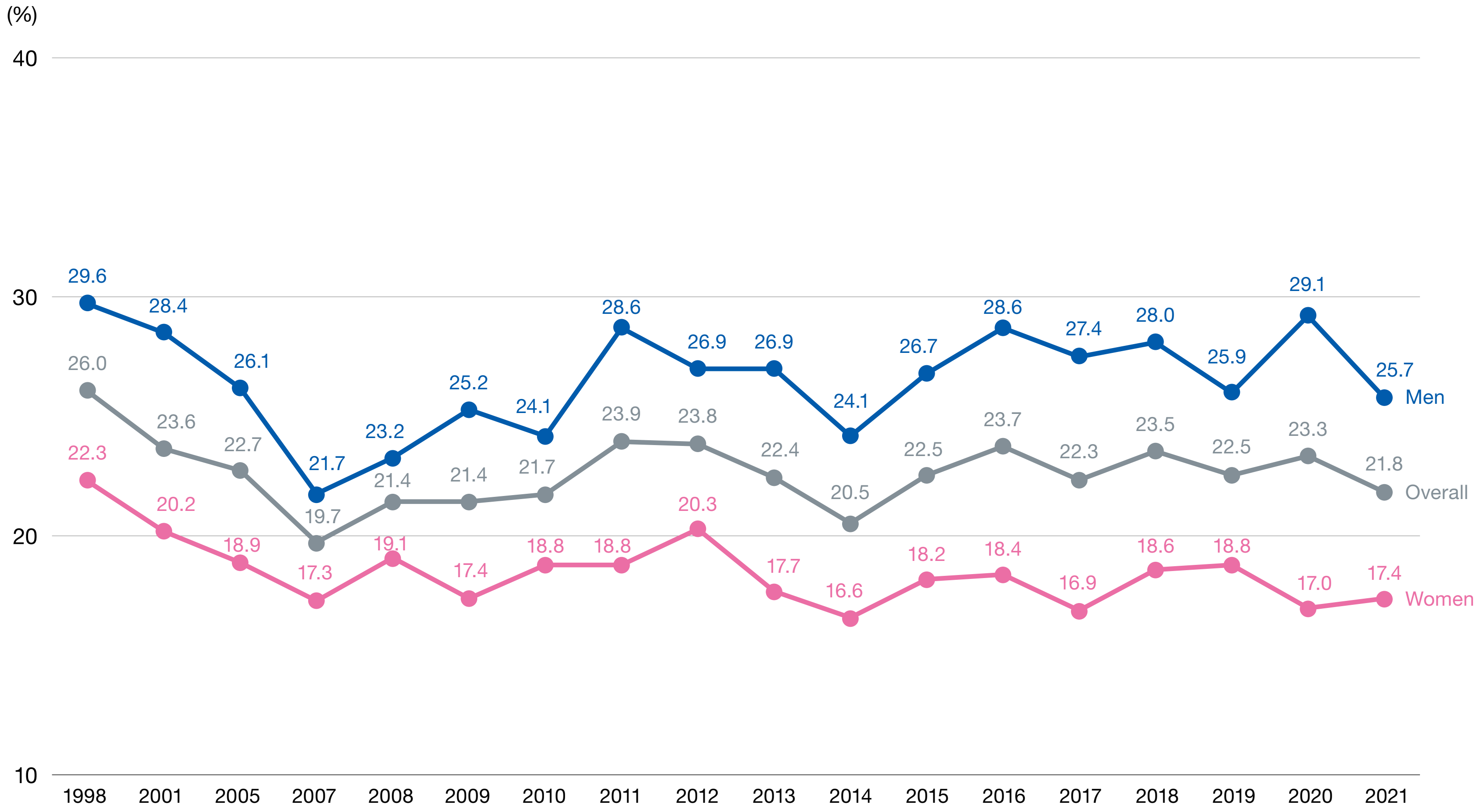

Data Source: Korea National Health and Nutrition Examination Survey 1998-2021 (directly age-standardized to the 2005 projected population)

Trends of Hypertension Prevalence

(Age 30+, Age-standardized)

	Crude	Age-standardized
Age 30+	**33** %	**27** %

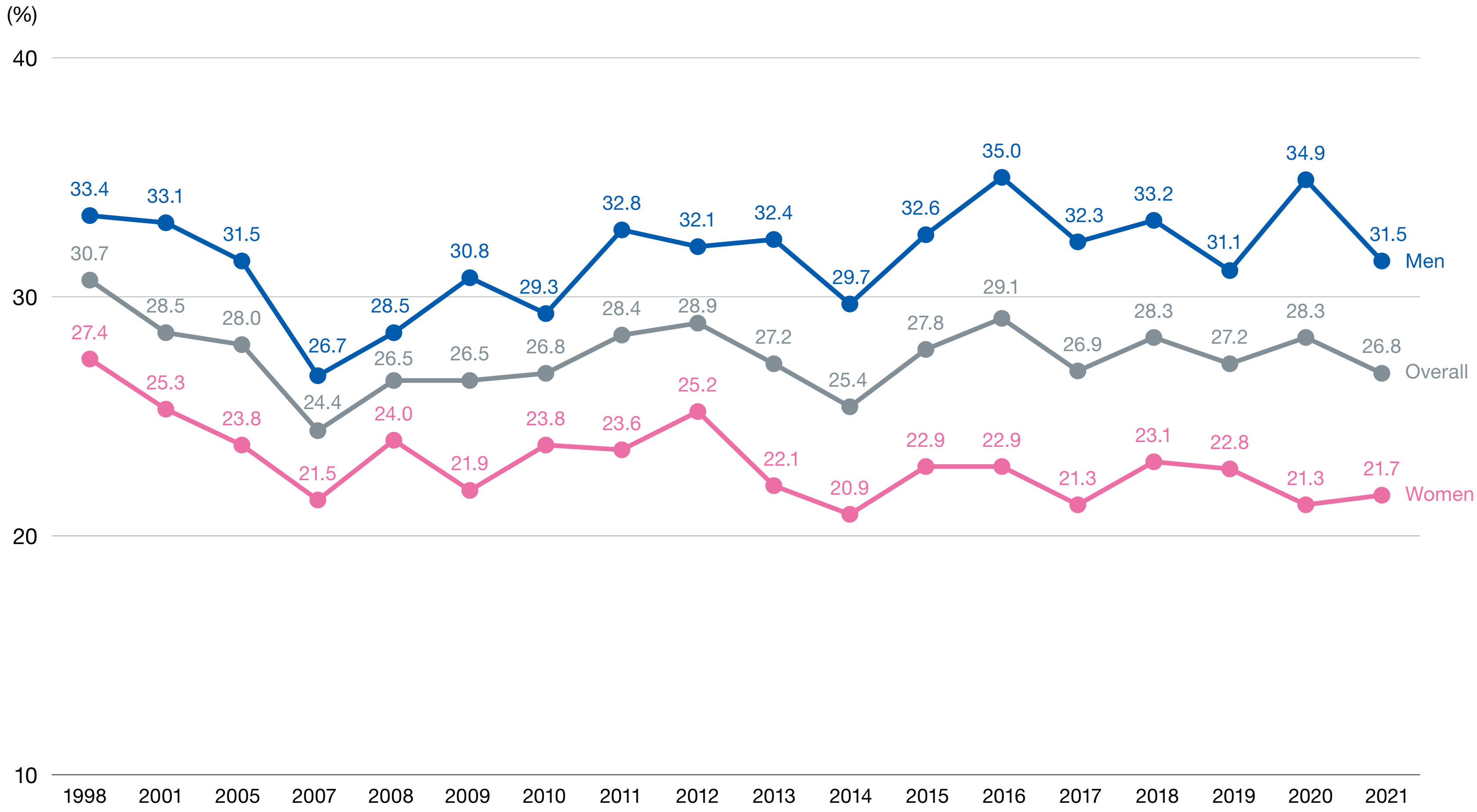

Data Source: Korea National Health and Nutrition Examination Survey 1998-2021 (directly age-standardized to the 2005 projected population)

Trends of Hypertension Prevalence by Sex and Age

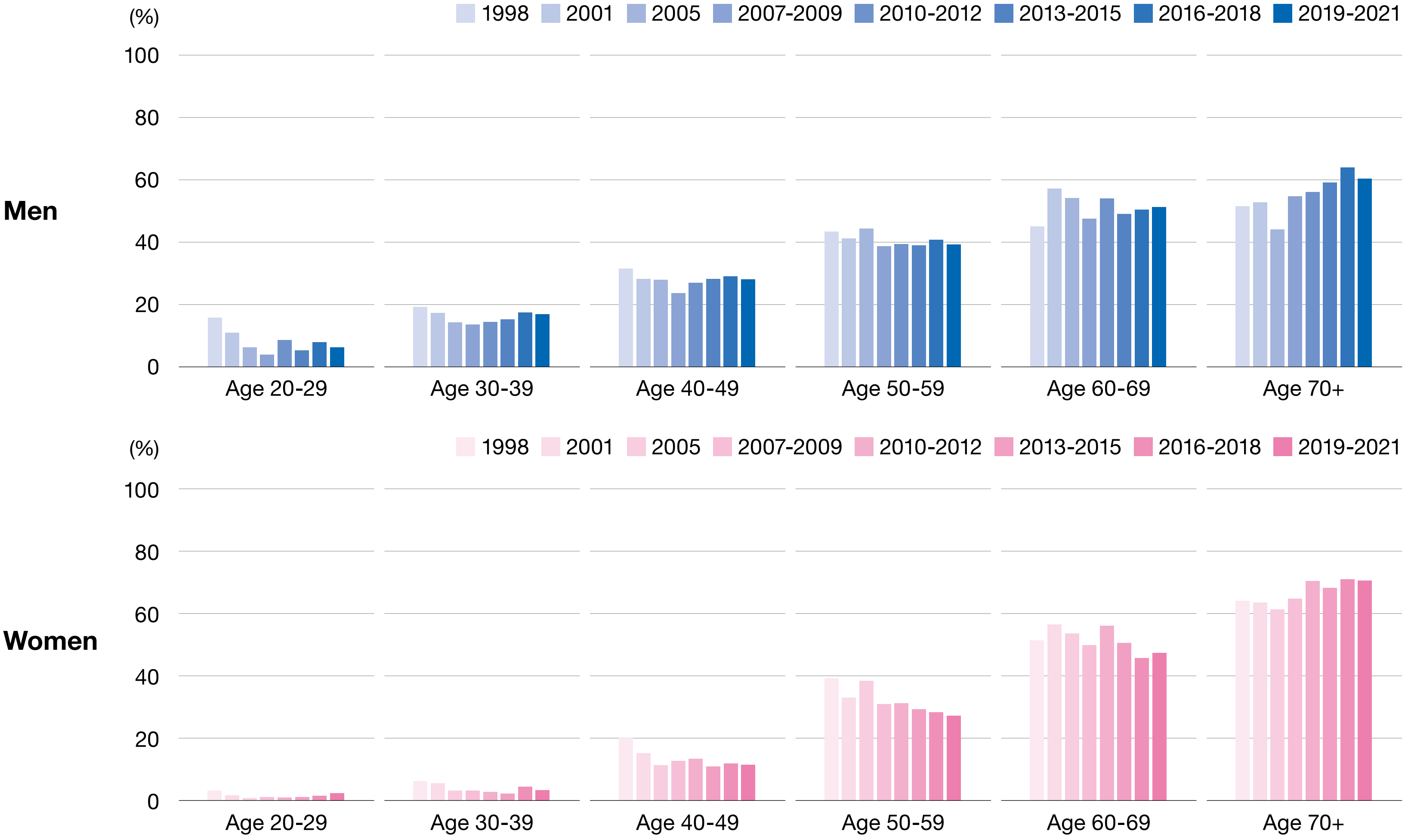

Data Source: Korea National Health and Nutrition Examination Survey 1998-2021

Trends of Number of People with Hypertension

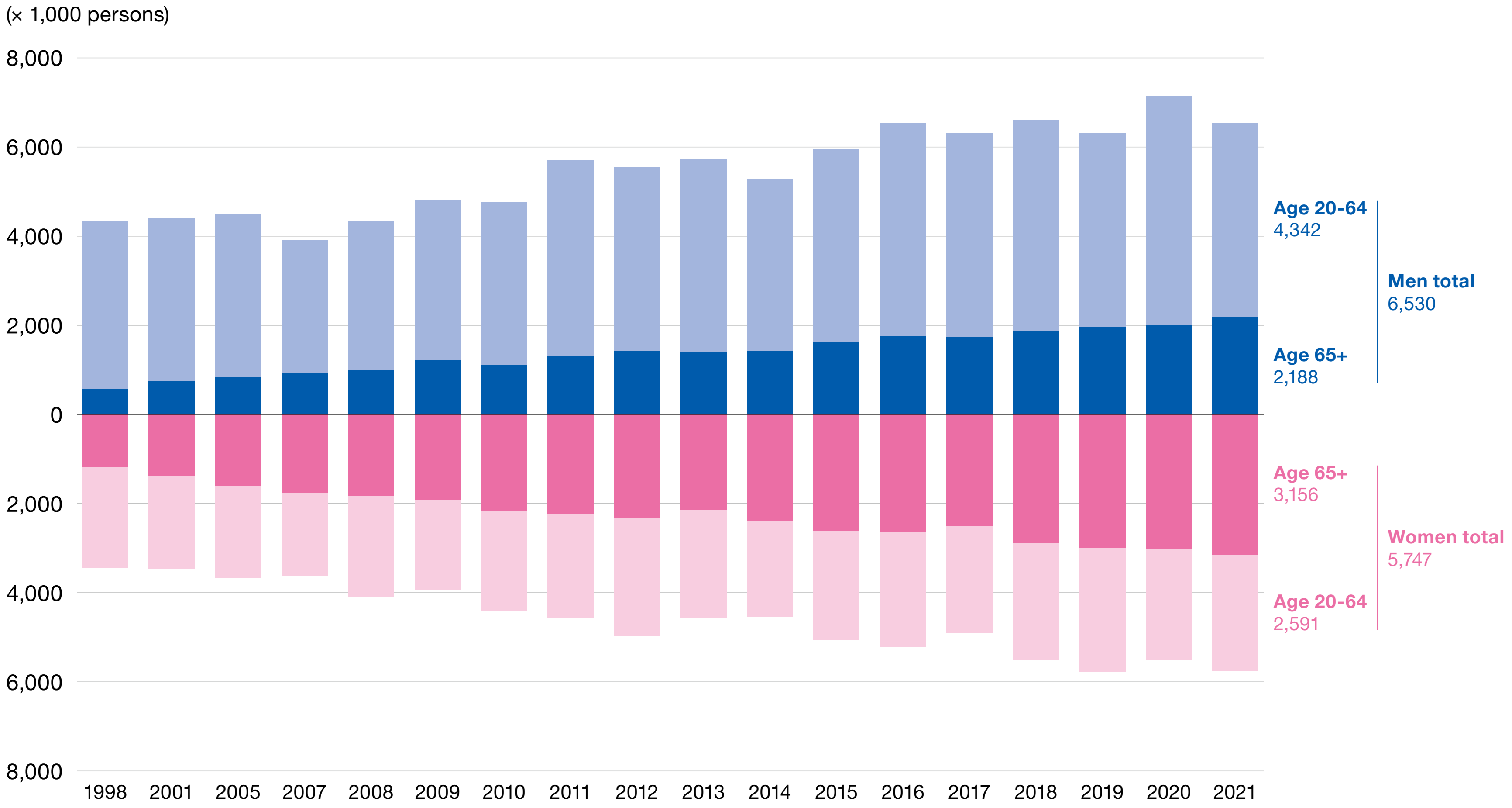

Data Source: Korea National Health and Nutrition Examination Survey 1998-2021

Trends of Hypertension Management

Trends of Awareness Rate by Sex and Age
Trends of Treatment Rate by Sex and Age
Trends of Control Rate by Sex and Age
Trends of Average Blood Pressure

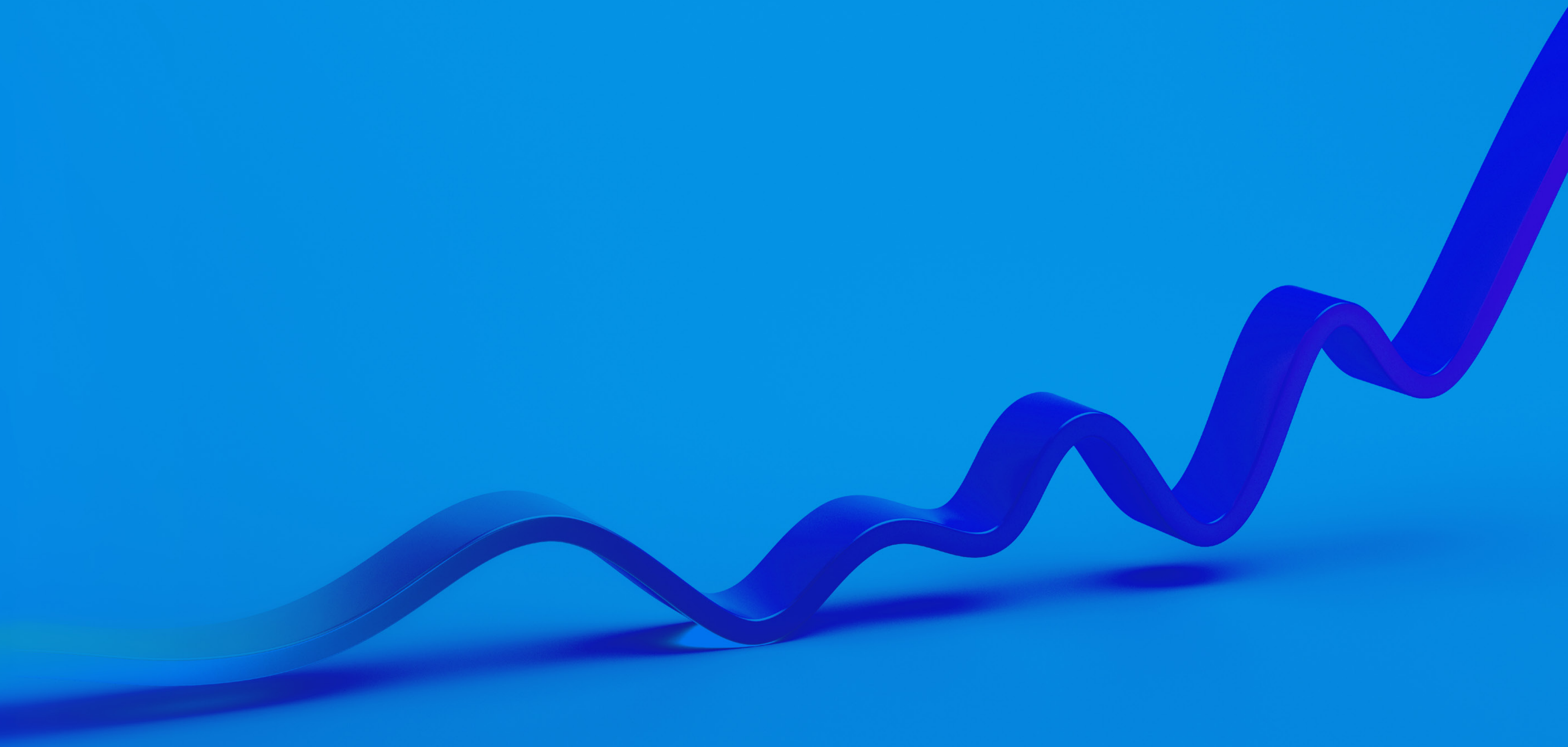

Trends of Awareness Rate by Sex and Age

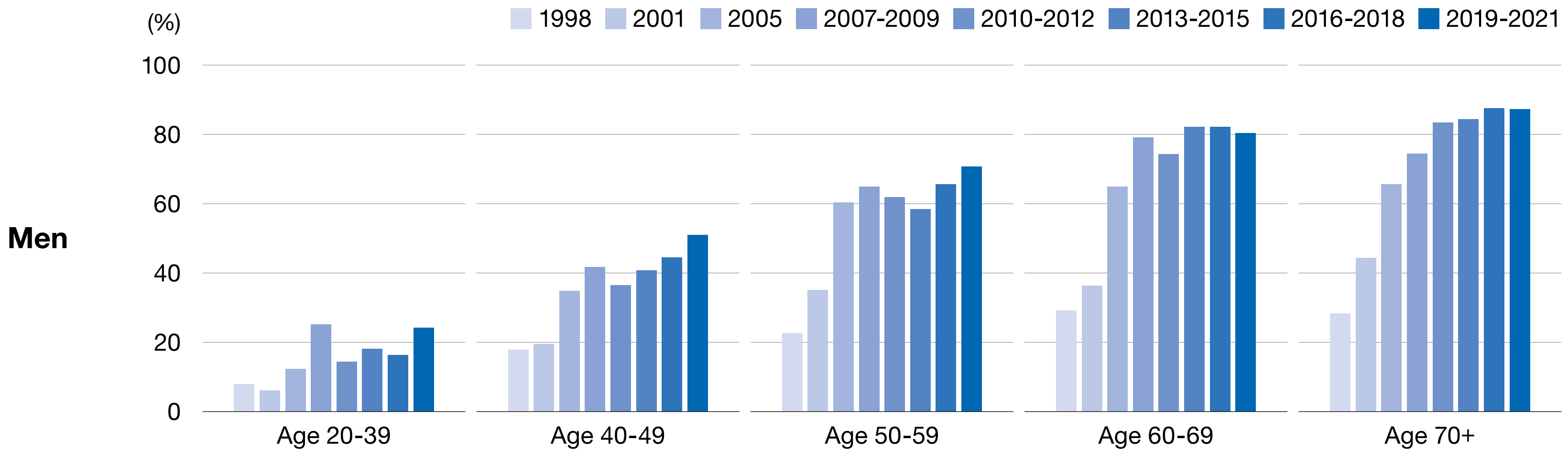

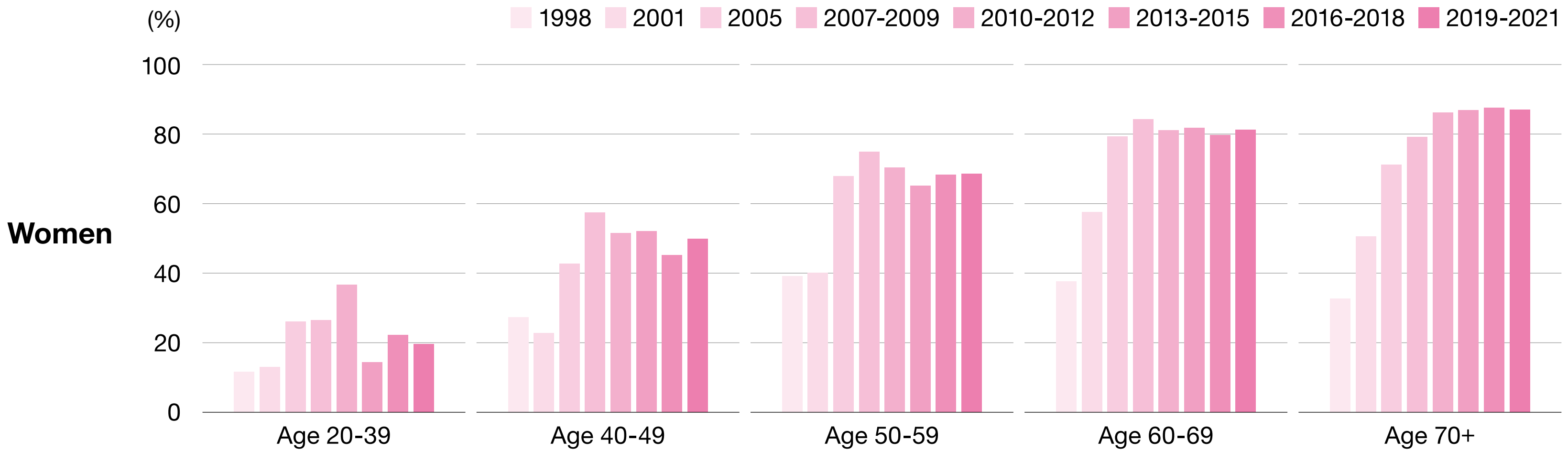

Data Source: Korea National Health and Nutrition Examination Survey 1998-2021

Trends of Treatment Rate by Sex and Age

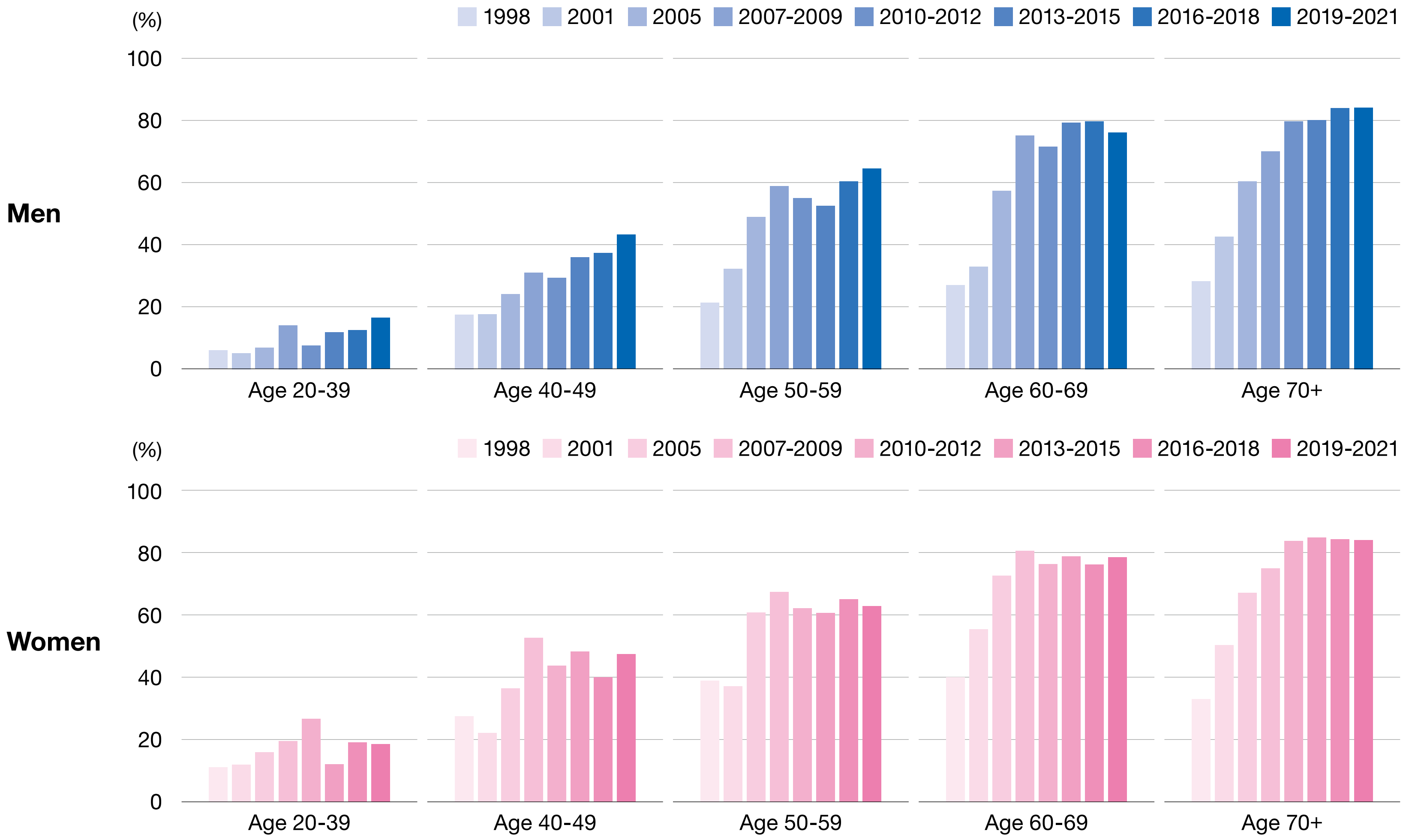

Data Source: Korea National Health and Nutrition Examination Survey 1998-2021

Trends of Control Rate by Sex and Age

(Among prevalent)

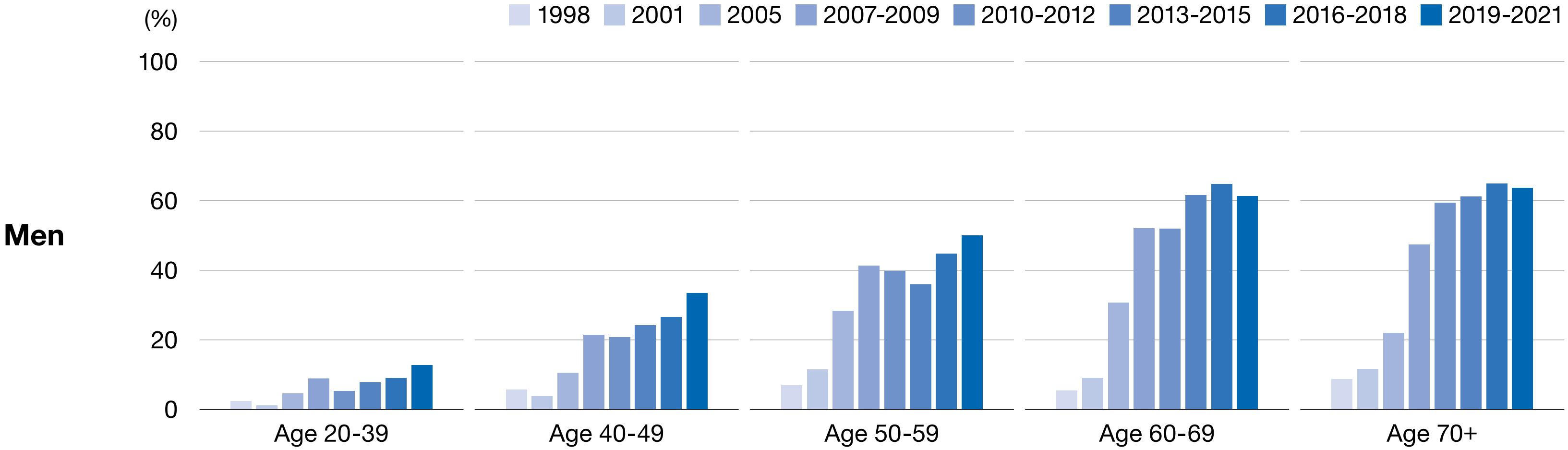

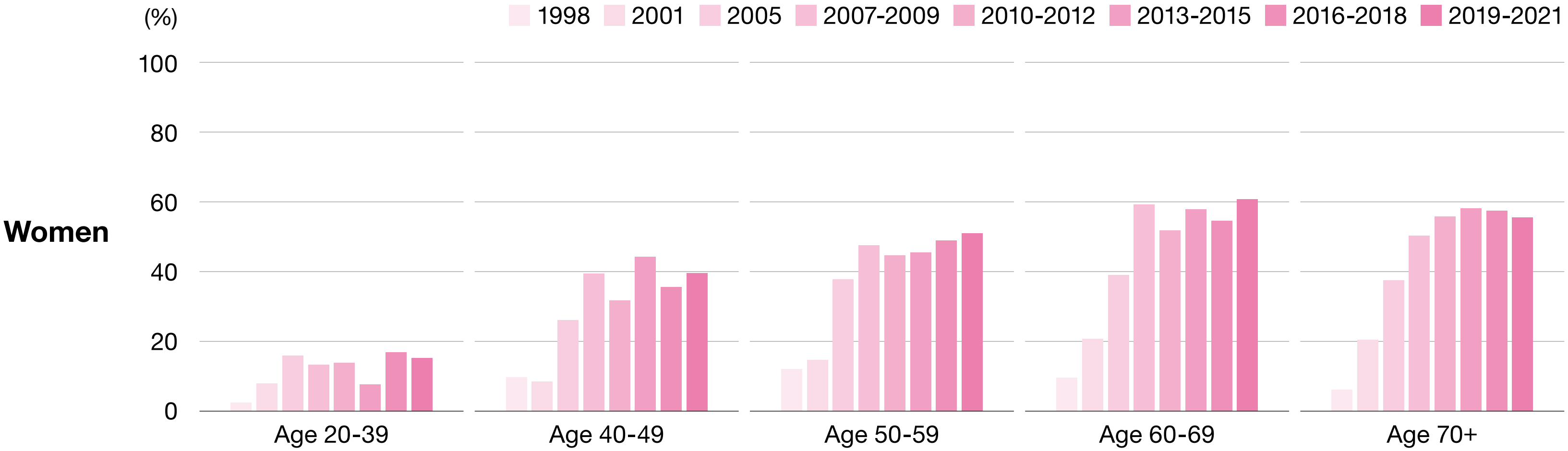

Data Source: Korea National Health and Nutrition Examination Survey 1998-2021

Trends of Control Rate by Sex and Age

(Among treated)

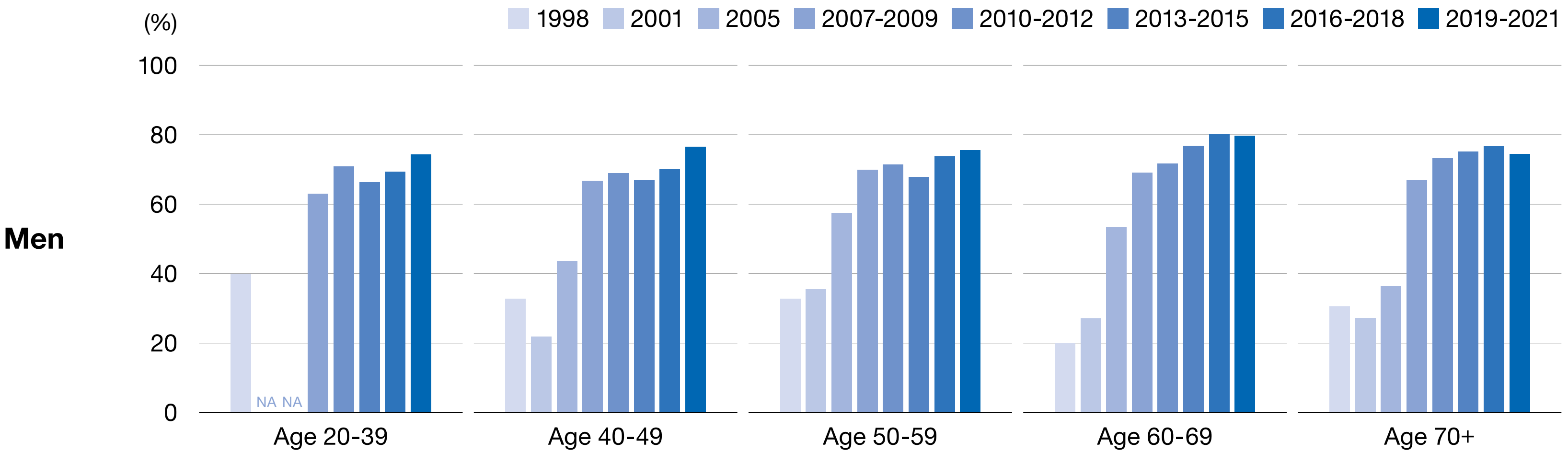

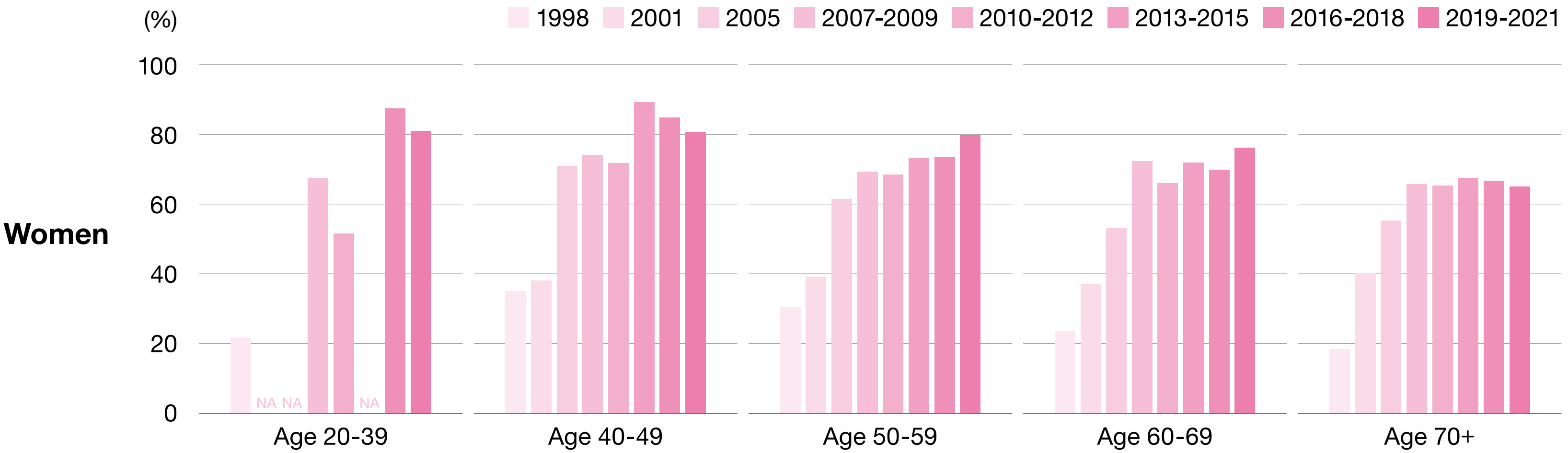

Data Source: Korea National Health and Nutrition Examination Survey 1998-2021

NA = Denominator less than 10

Trends of Average Blood Pressure

(Among prevalent)

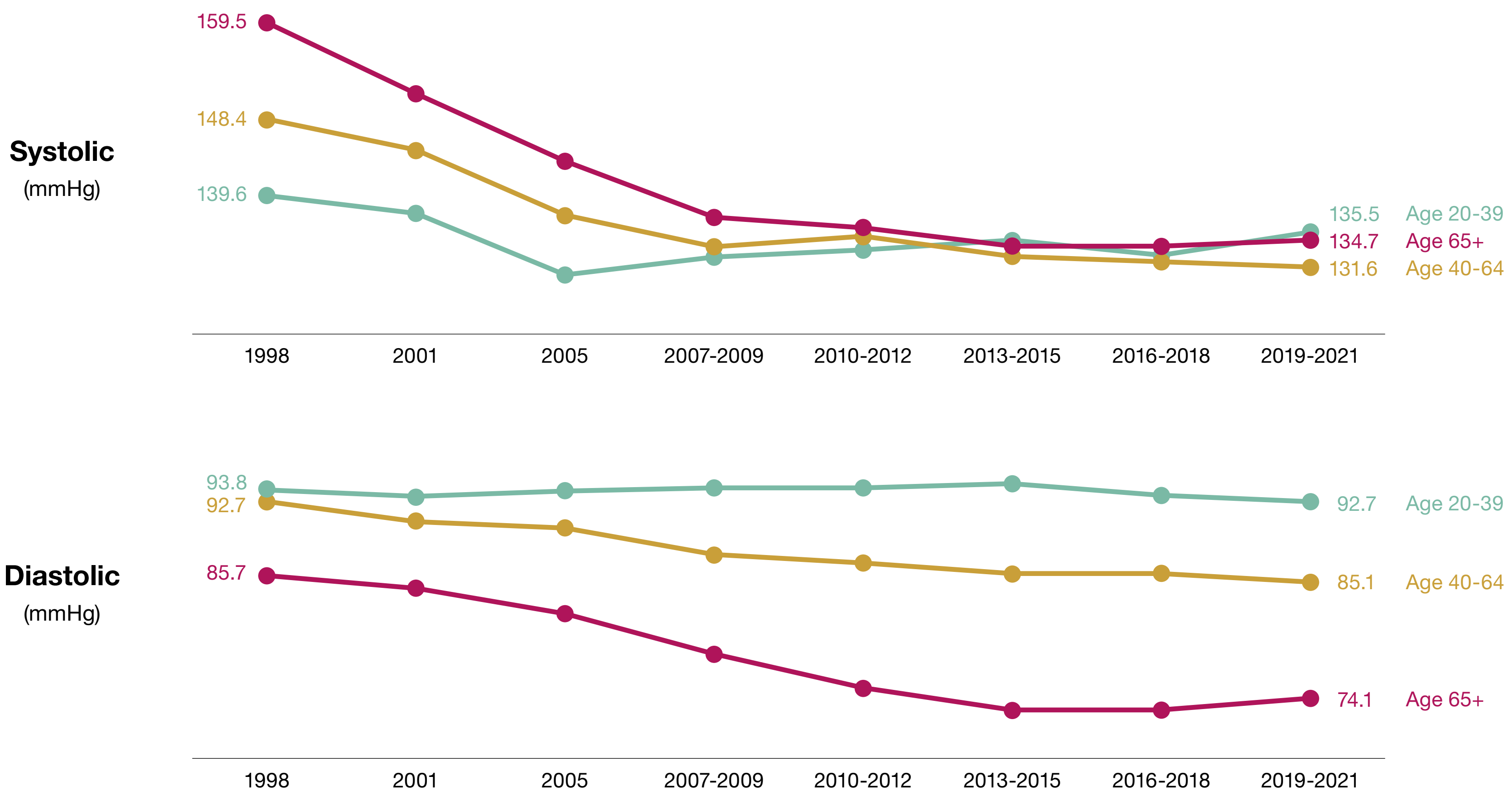

Data Source: Korea National Health and Nutrition Examination Survey 1998-2021

Trends of Average Blood Pressure

(Among treated)

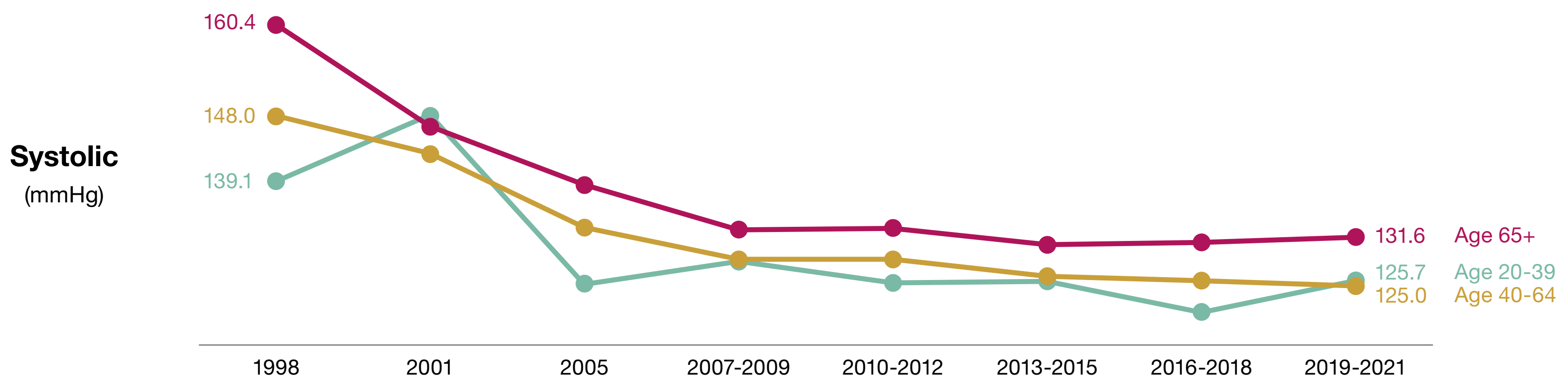

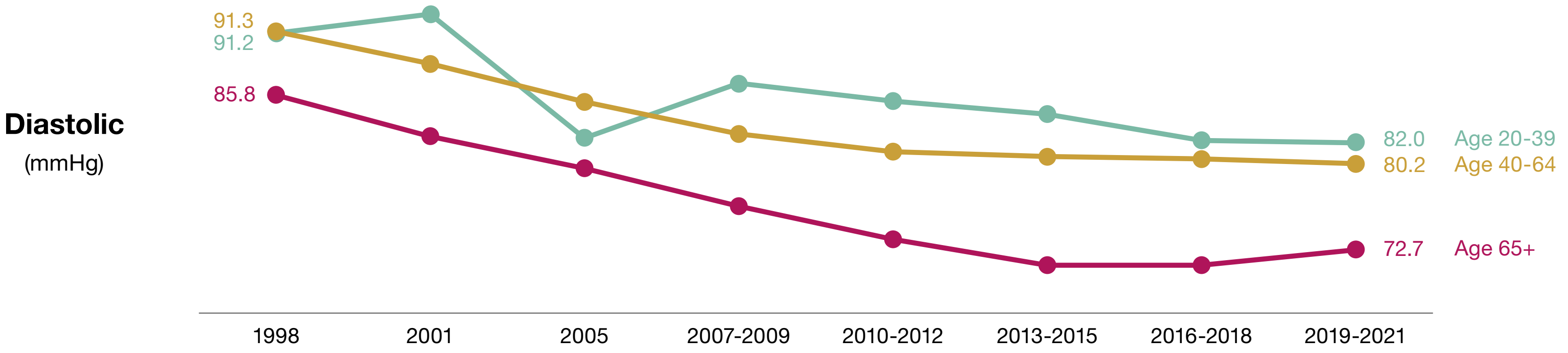

Data Source: Korea National Health and Nutrition Examination Survey 1998-2021

Healthcare Utilization for Hypertension

Trends of Healthcare Utilization for Hypertension
Trends of Co-treatment for Dyslipidemia and Diabetes
Trends of Antihypertensive Medication Use
Composition of Antihypertensive Treatment
Antihypertensive Medication Use By Co-treatment Status

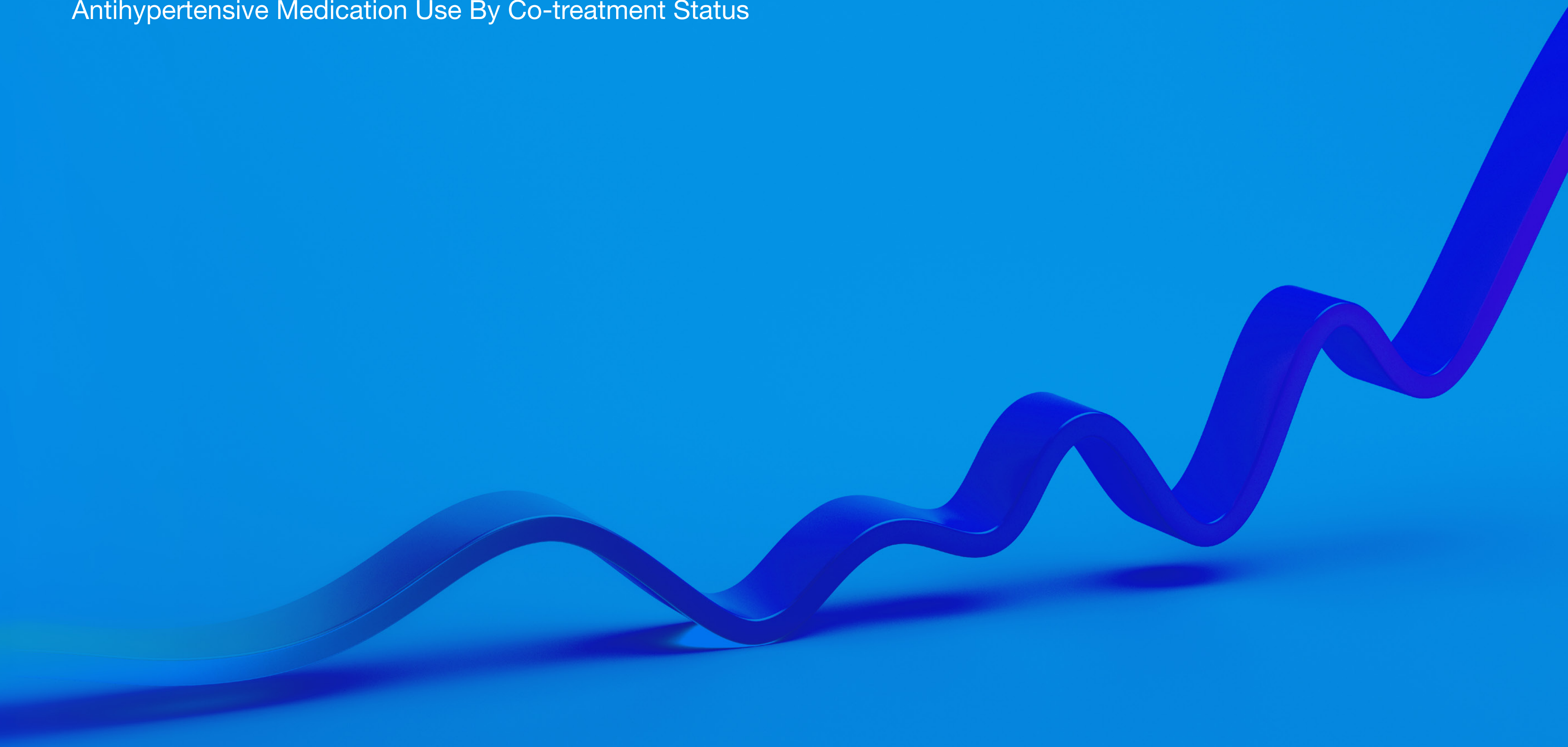

Trends of Healthcare Utilization for Hypertension

(Age 20+)

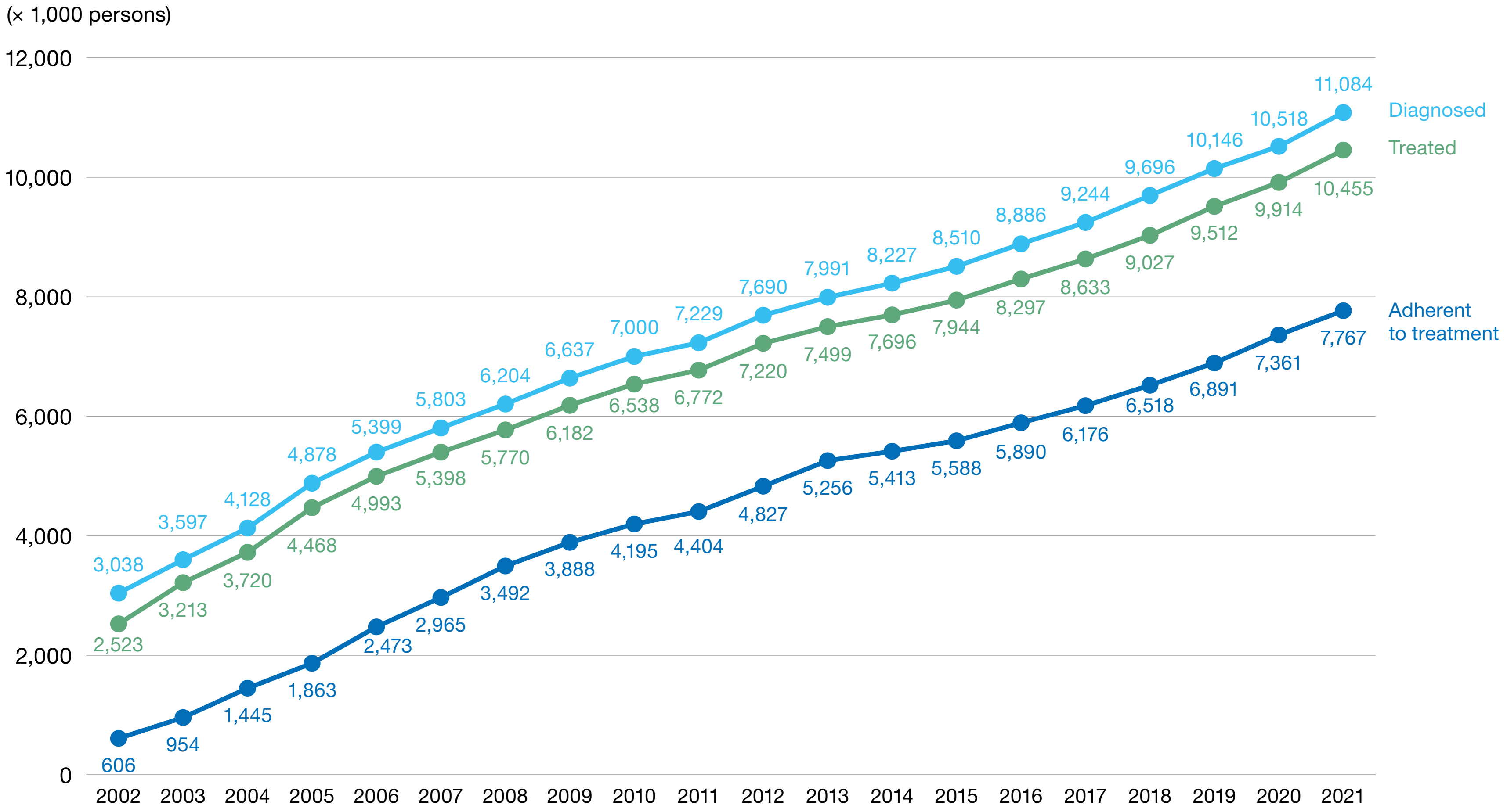

Data Source: Korea National Health Insurance Big Data 2002-2021

Trends of Co-treatment for Dyslipidemia and Diabetes

(Age 20+)

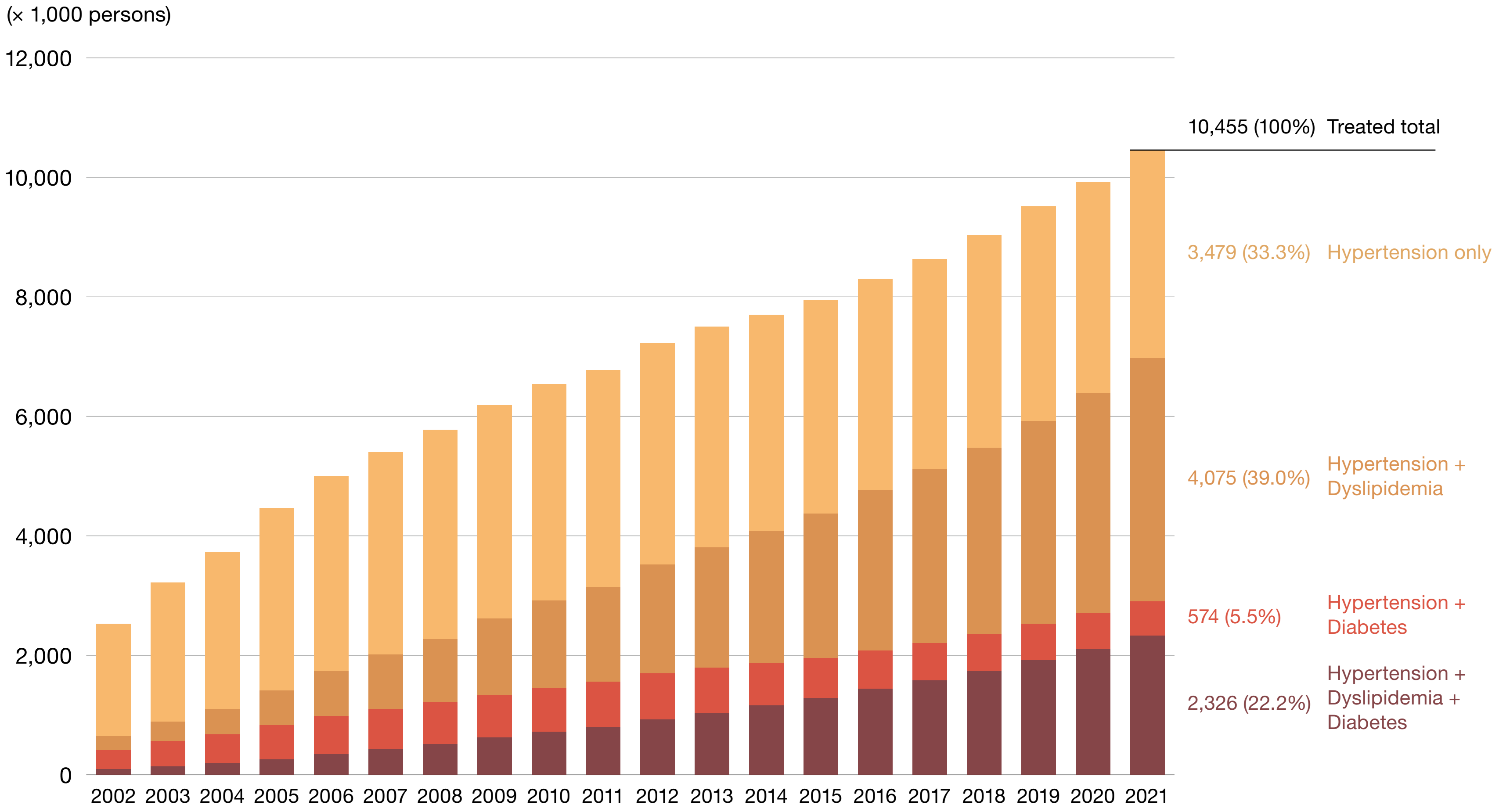

Data Source: Korea National Health Insurance Big Data 2002-2021

Trends of Antihypertensive Medication Use

(Age 20+)

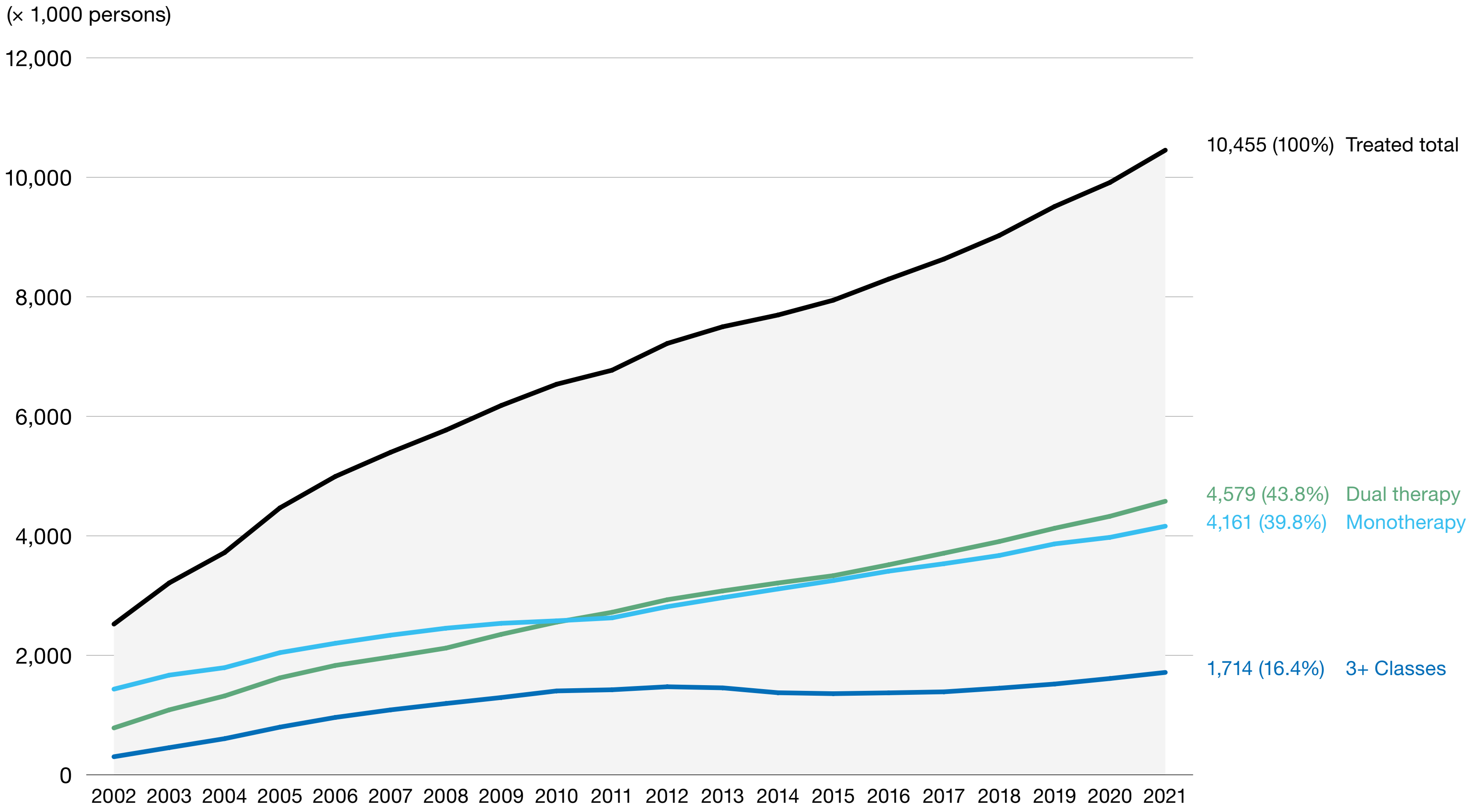

Data Source: Korea National Health Insurance Big Data 2002-2021

Trends of Antihypertensive Medication Use

(Age 20+)

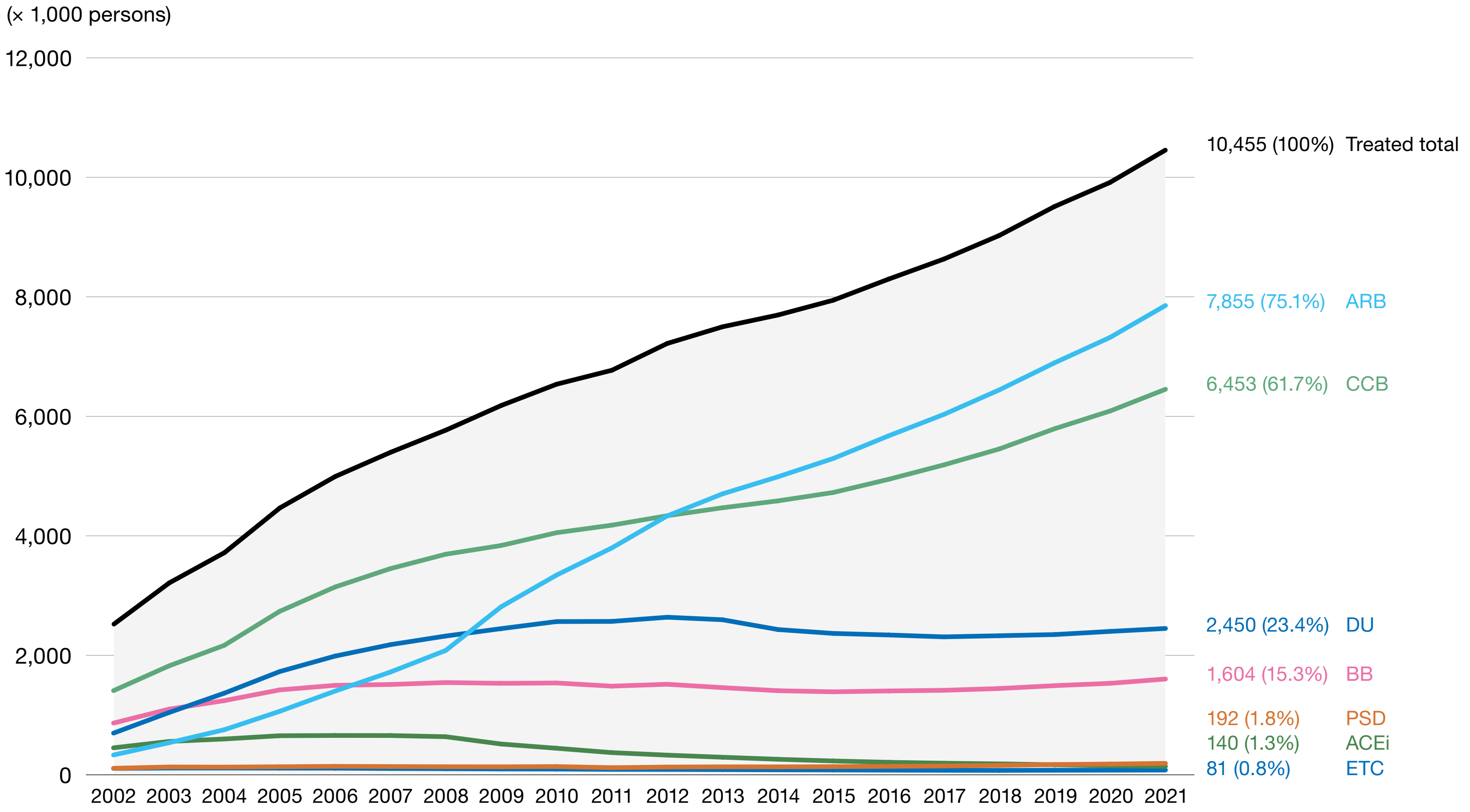

Data Source: Korea National Health Insurance Big Data 2002-2021

Composition of Antihypertensive Treatment

(Age 20+)

Monotherapy

Among 4.2 million, %

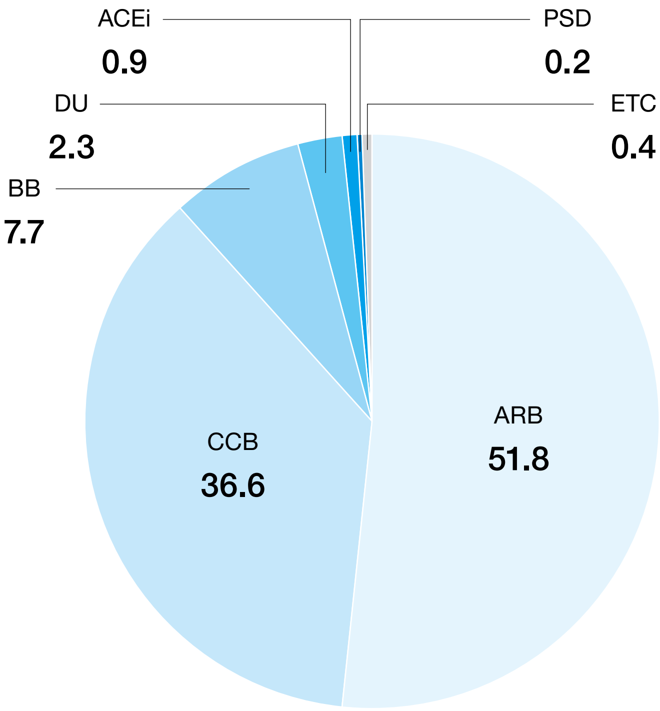

Dual therapy

Among 4.6 million, %

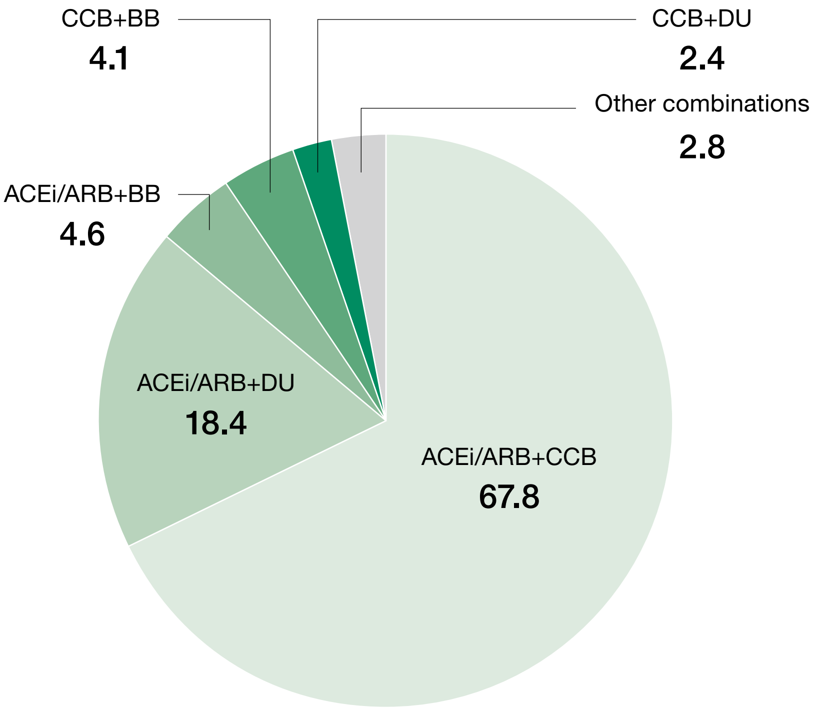

Data Source: Korea National Health Insurance Big Data 2021

Composition of Antihypertensive Treatment

(Age 20+)

Triple therapy

Among 1.4 million, %

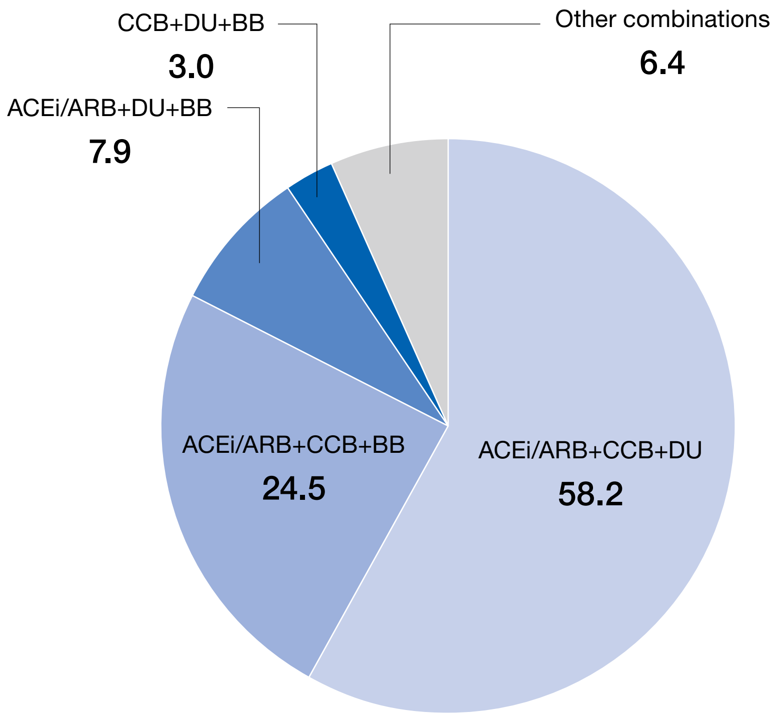

Quadruple therapy

Among 0.3 million, %

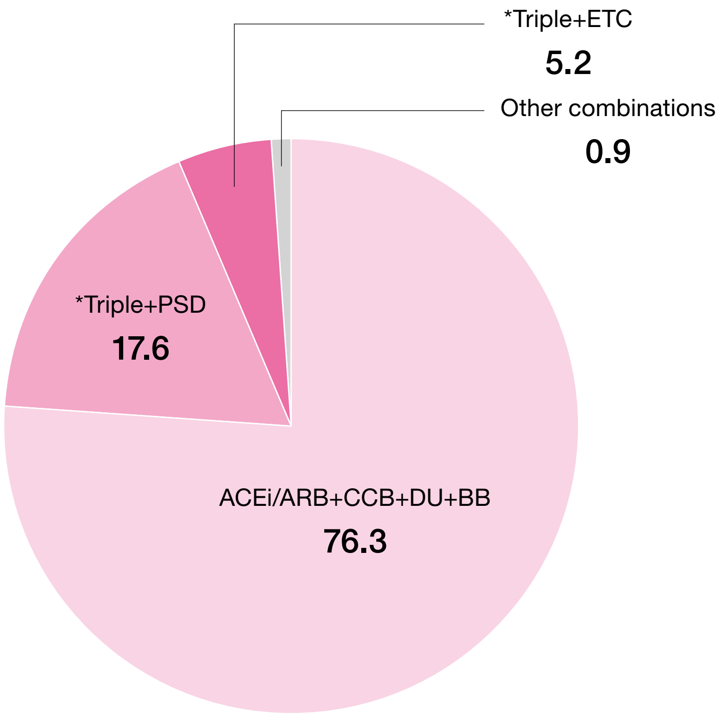

Data Source: Korea National Health Insurance Big Data 2021

*Triple = Three of the ACEi/ARB, CCB, DU, or BB

Antihypertensive Medication Use By Co-treatment Status

(Age 20+)

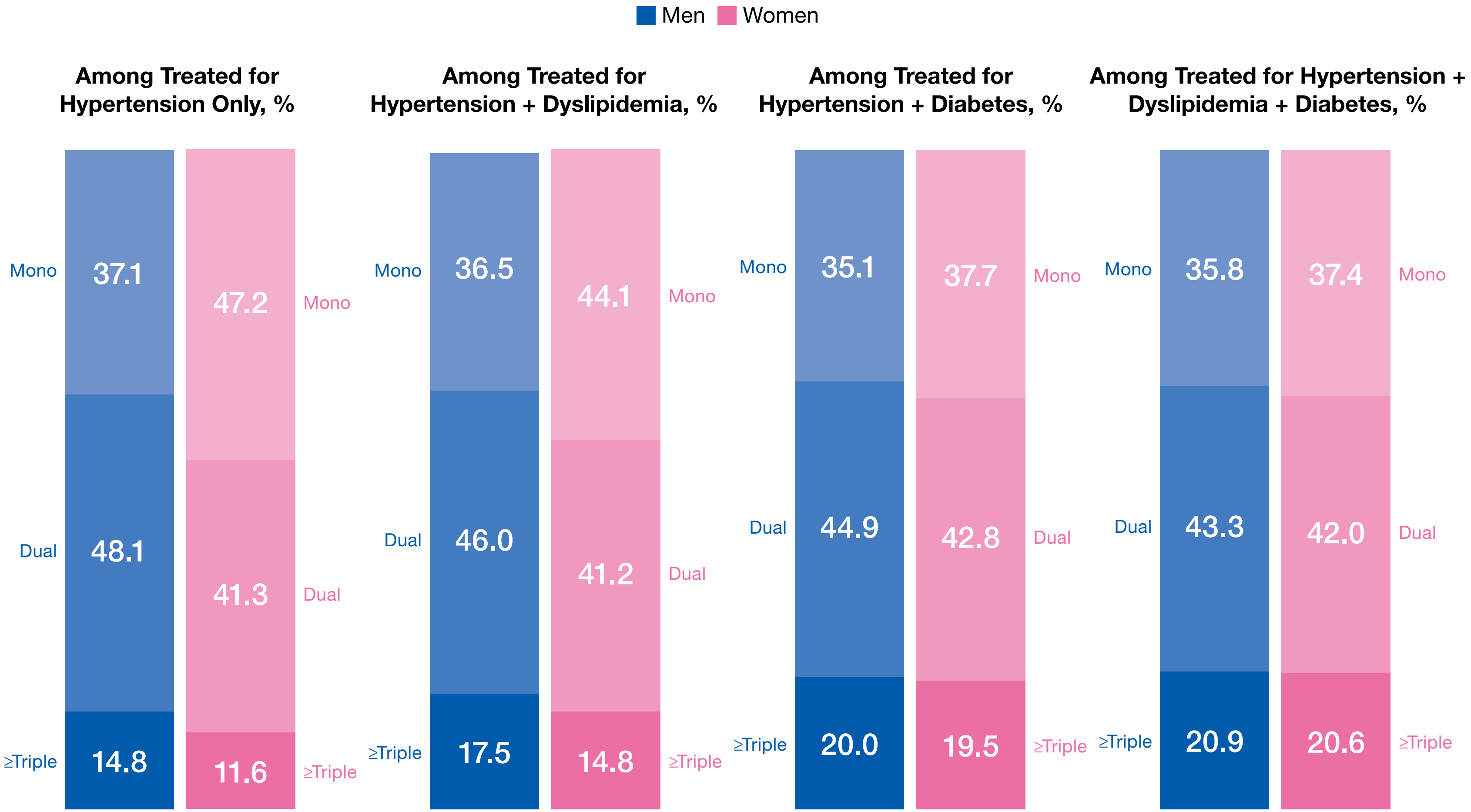

Data Source: Korea National Health Insurance Big Data 2021

Antihypertensive Medication Use By Co-treatment Status

(Age 20+)

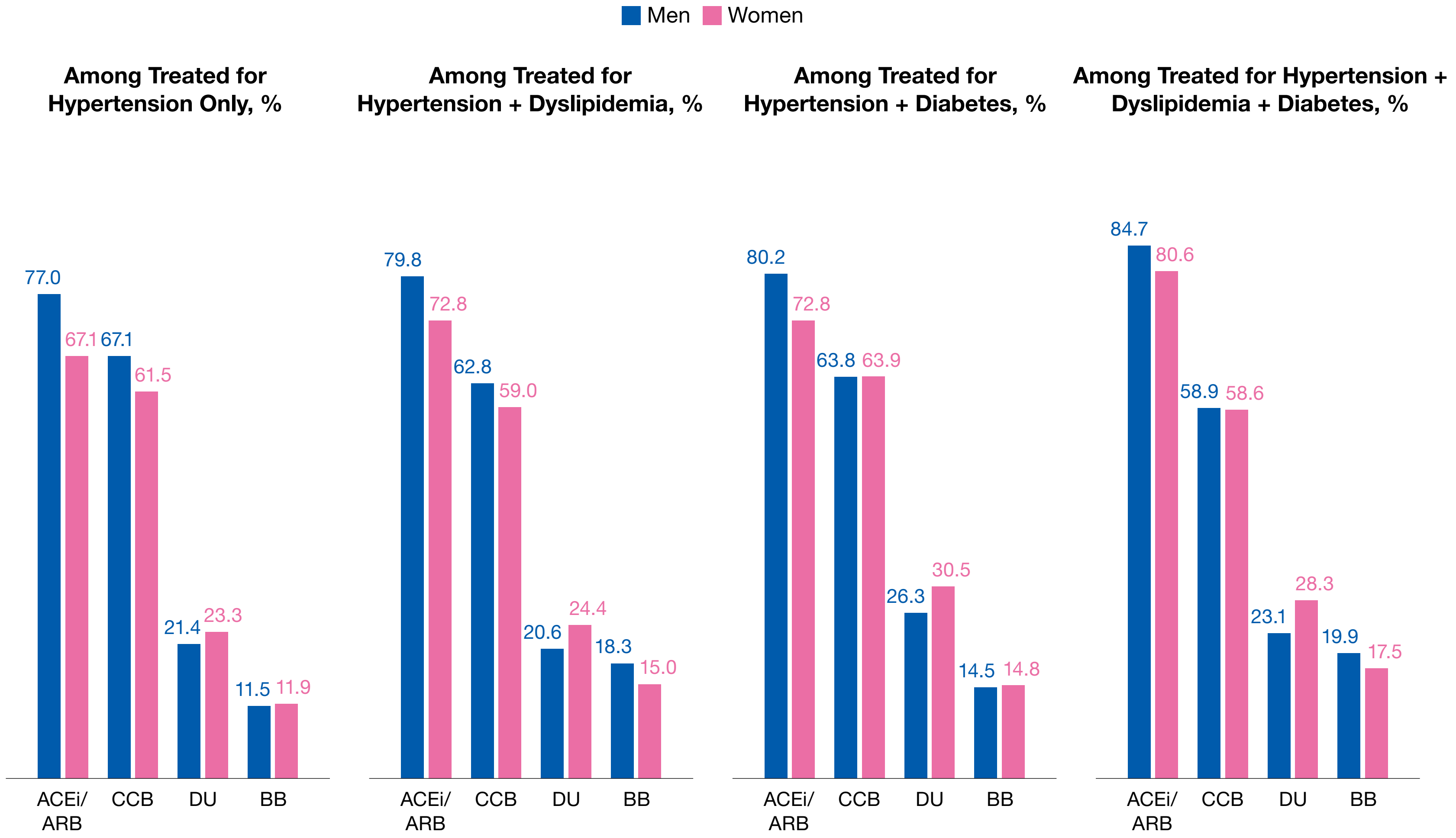

Data Source: Korea National Health Insurance Big Data 2021

Hypertension in Special Populations

Trends of Blood Pressure Control in Special Populations
Trends of Blood Pressure Control in the Elderly (Age 65+)
Trends of Blood Pressure Control in the Elderly (Age 80+)
Trends of Blood Pressure Control in People with Diabetes
Trends of Blood Pressure Control in People with Obesity
Trends of Blood Pressure Control in People with Chronic Kidney Disease
Trends of Blood Pressure Control in People with High-risk Hypertension

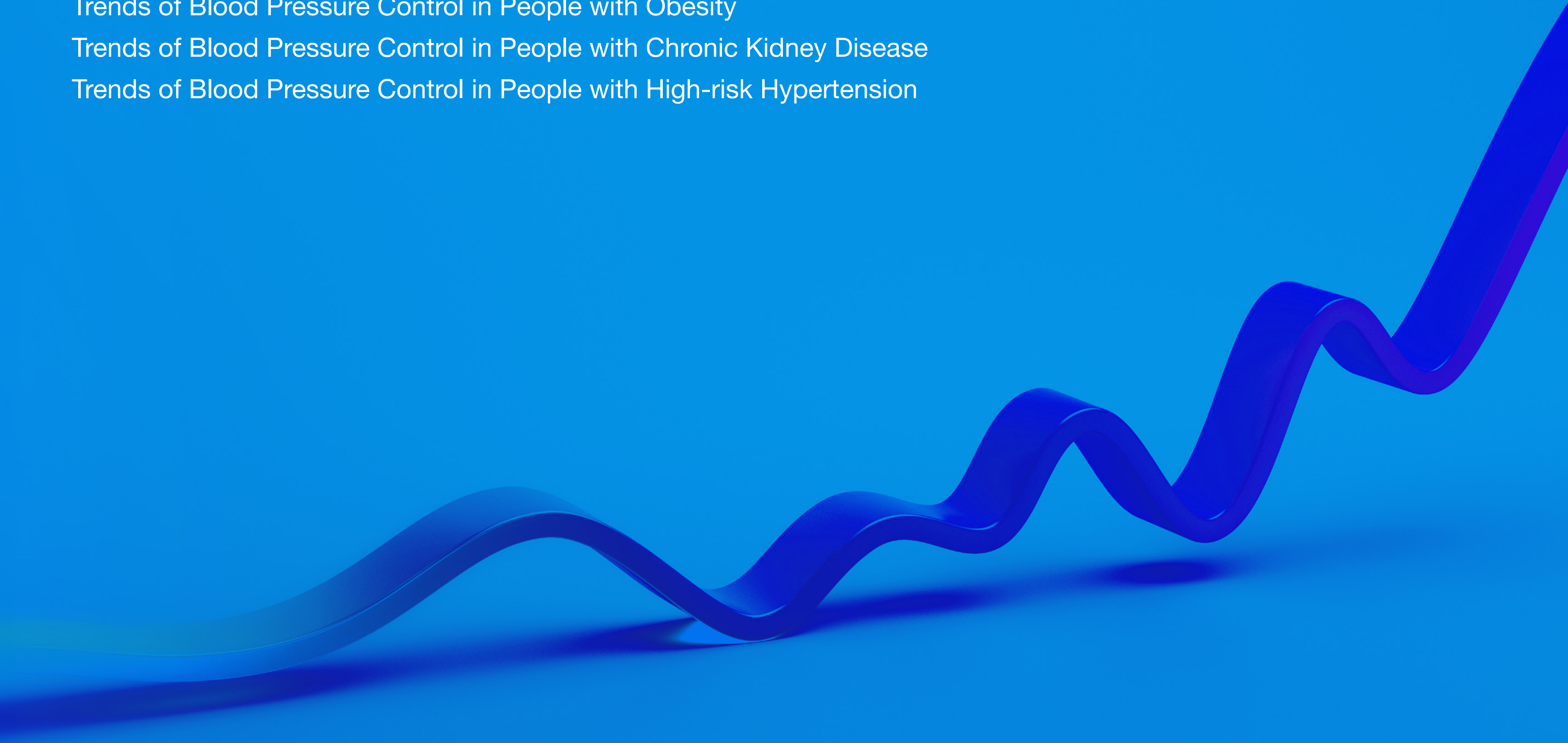

Trends of Blood Pressure Control in Special Populations

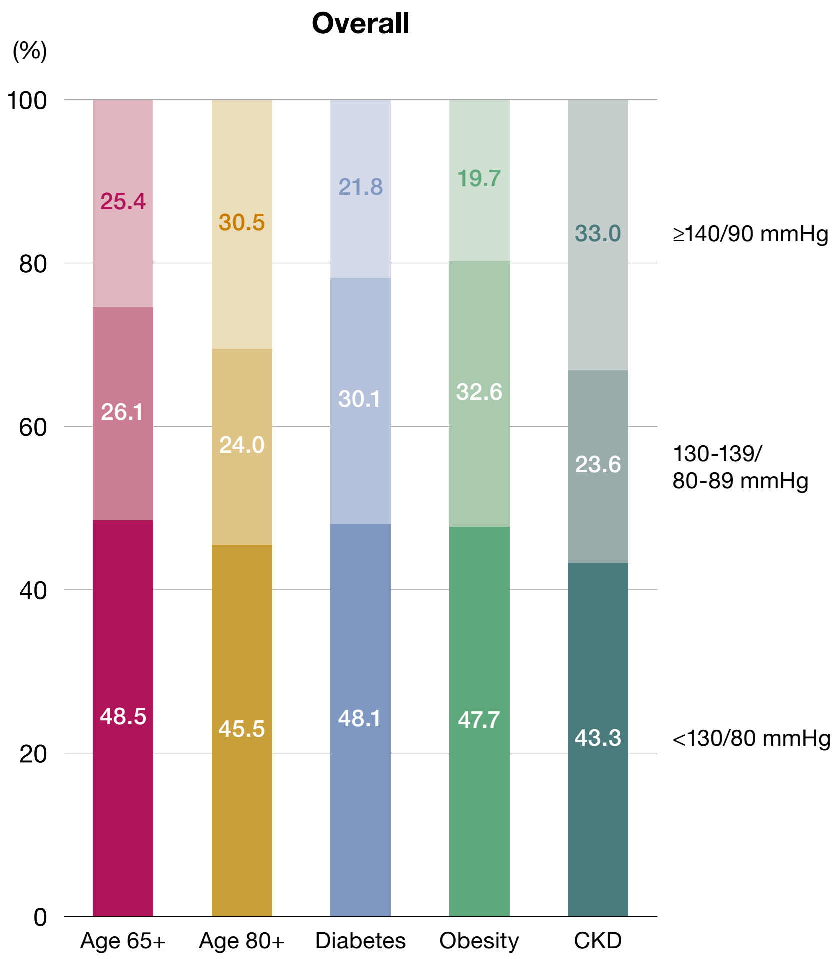

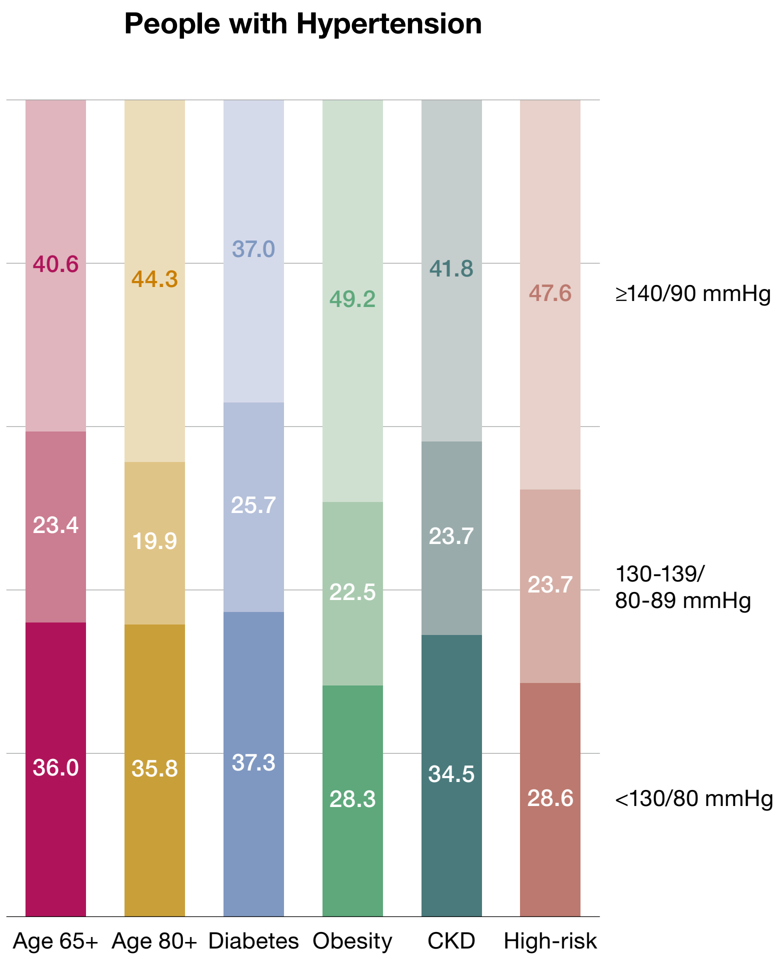

Data Source: Korea National Health and Nutrition Examination Survey 2019-2021

Trends of Blood Pressure Control in the Elderly (Age 65+)

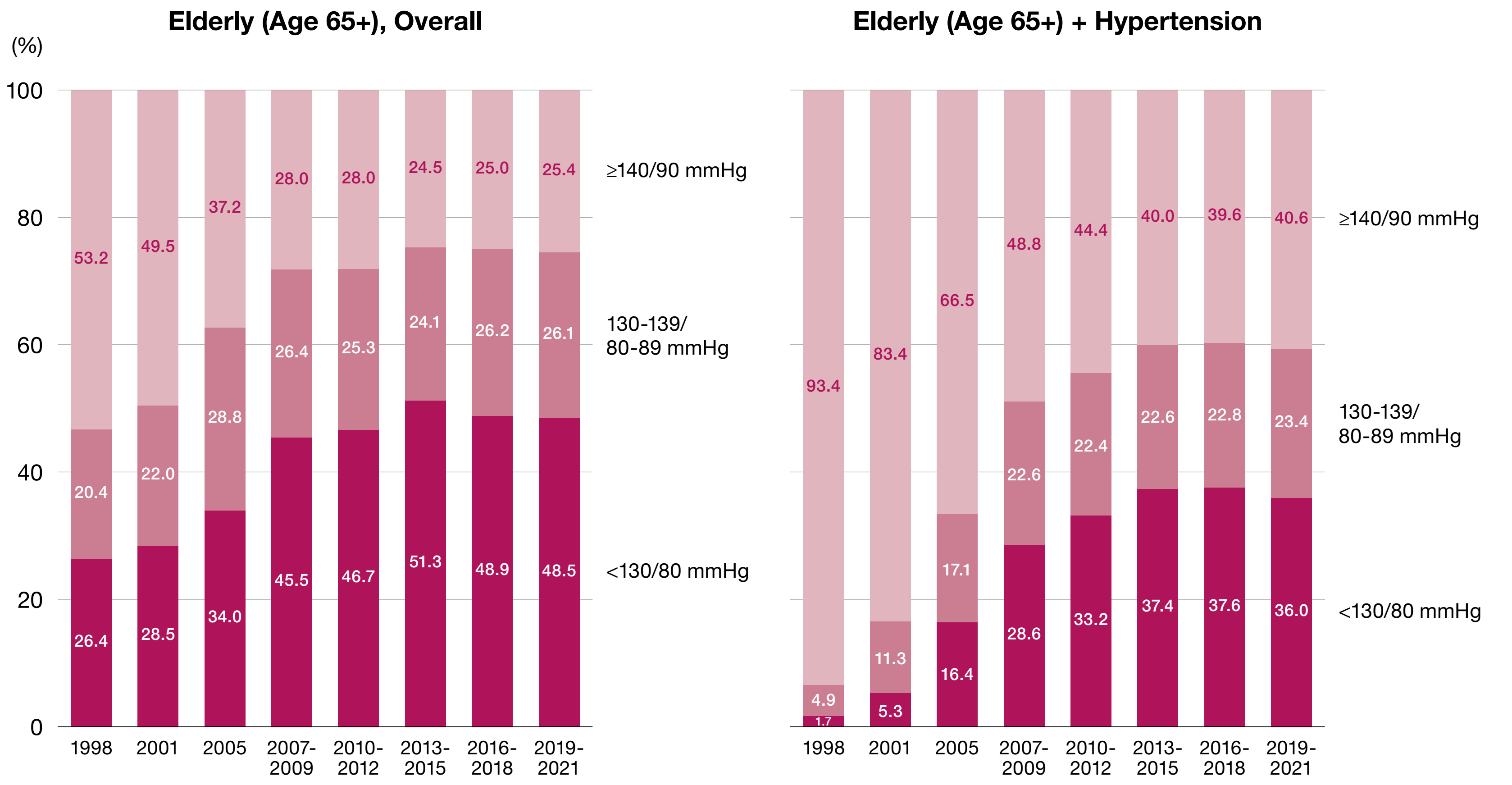

Data Source: Korea National Health and Nutrition Examination Survey 1998-2021

Trends of Blood Pressure Control in the Elderly (Age 65+)

Elderly (Age 65+), Overall

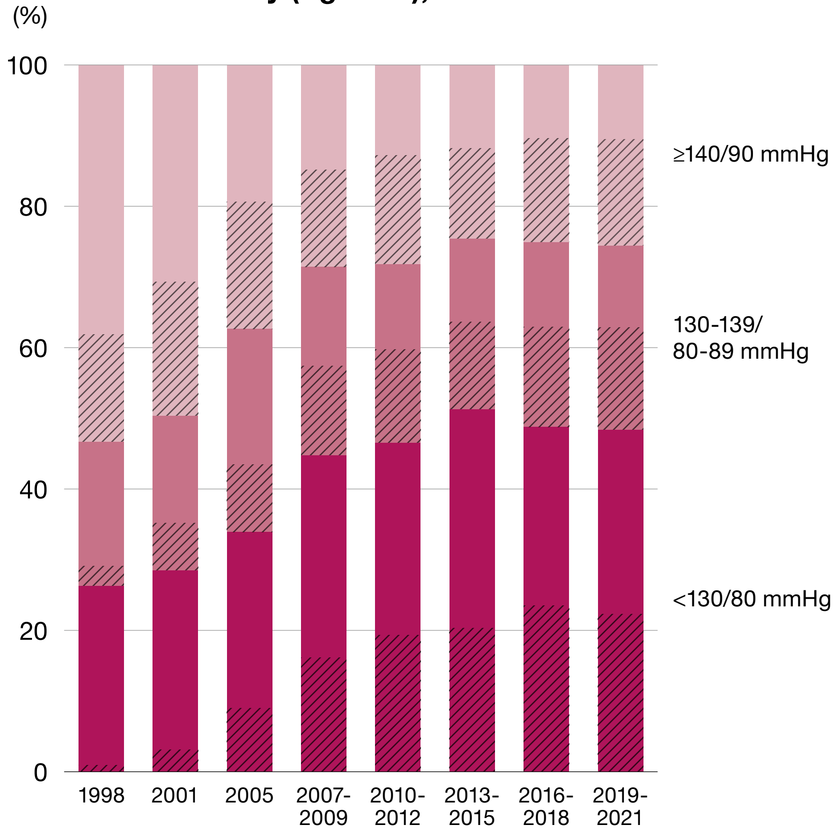

Elderly (Age 65+) + Treated for Hypertension

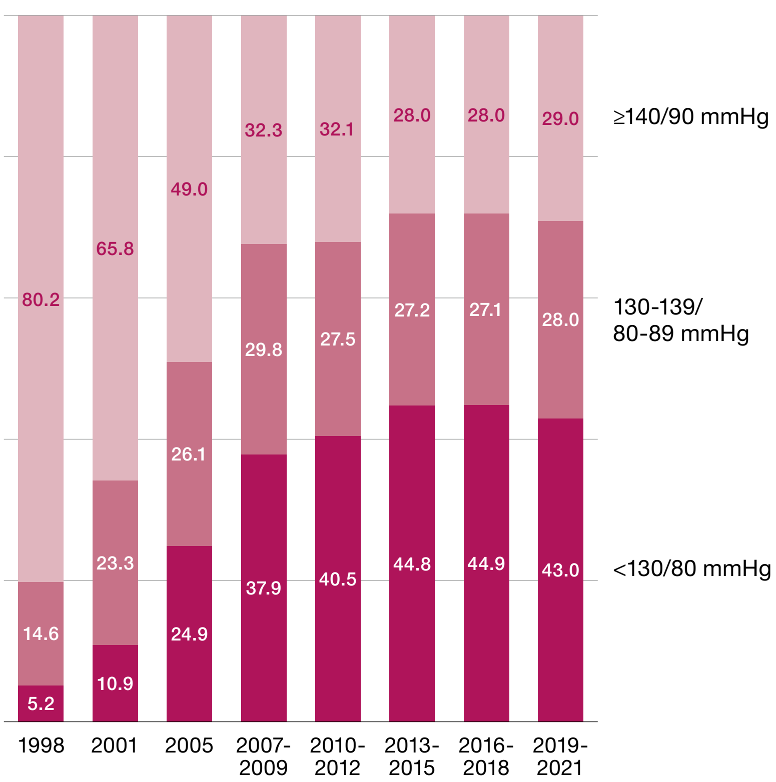

Shaded area: People treated for hypertension

Data Source: Korea National Health and Nutrition Examination Survey 1998-2021

Trends of Blood Pressure Control in the Elderly (Age 80+)

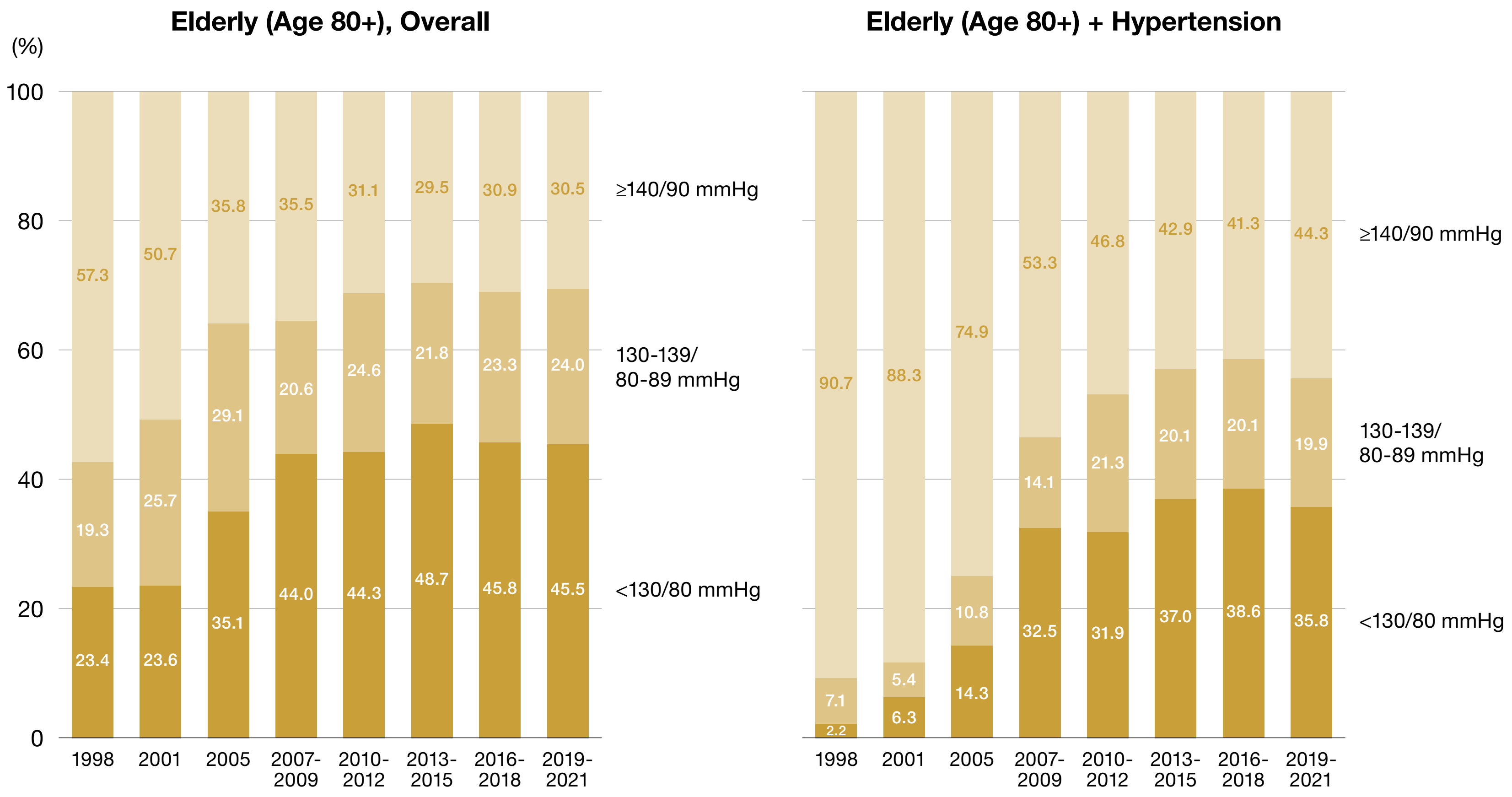

Data Source: Korea National Health and Nutrition Examination Survey 1998-2021

Trends of Blood Pressure Control in the Elderly (Age 80+)

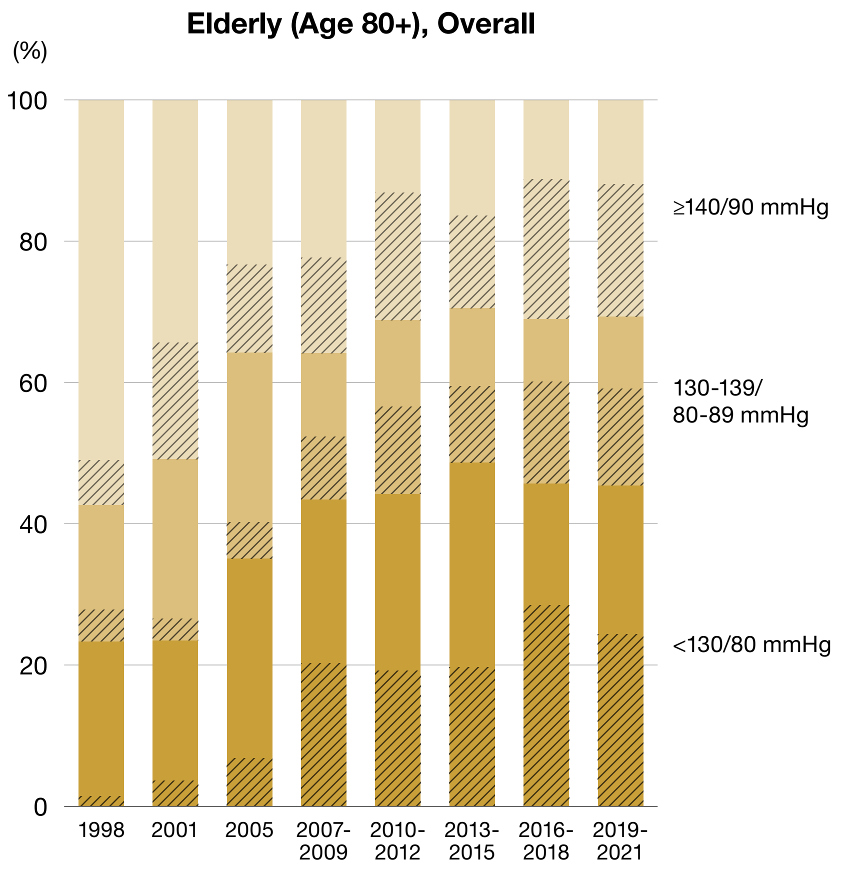

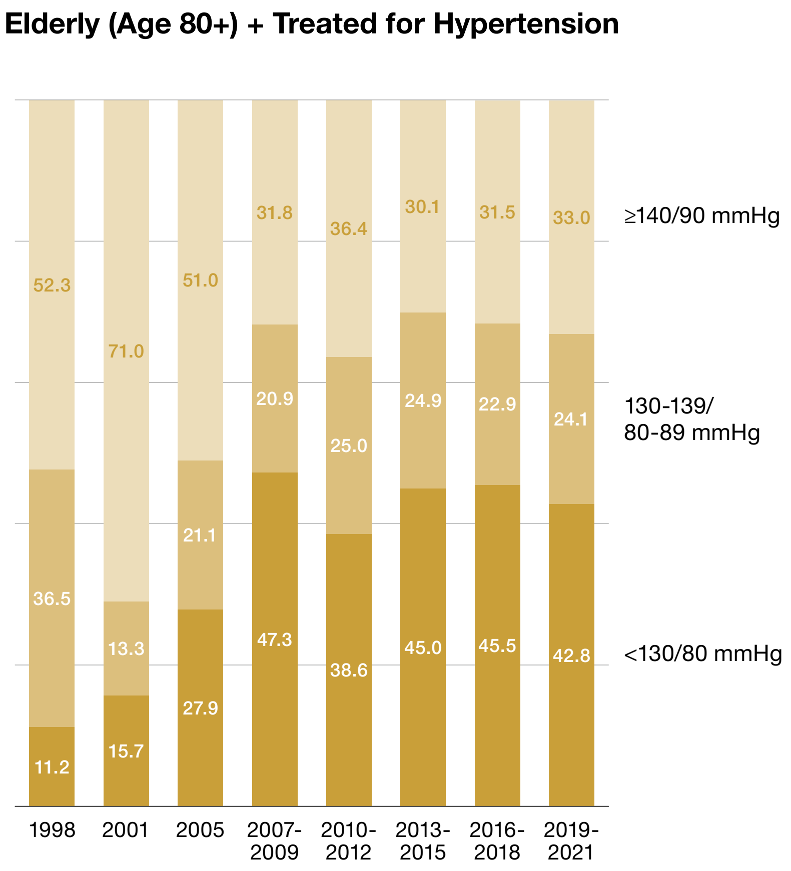

Shaded area: People treated for hypertension

Data Source: Korea National Health and Nutrition Examination Survey 1998-2021

Trends of Blood Pressure Control in People with Diabetes

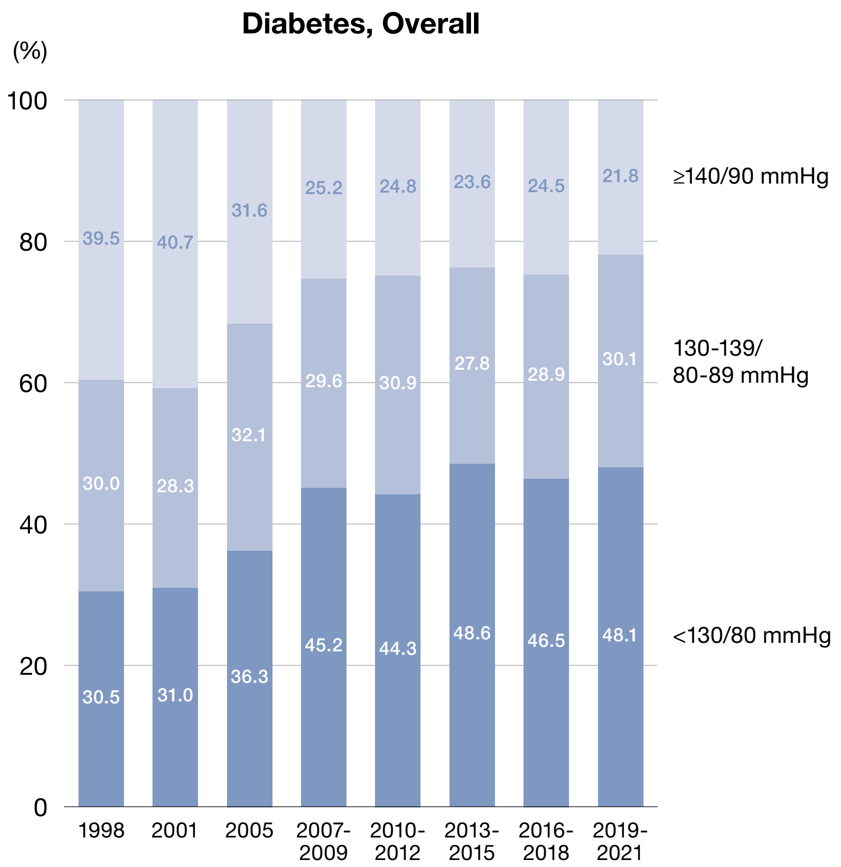

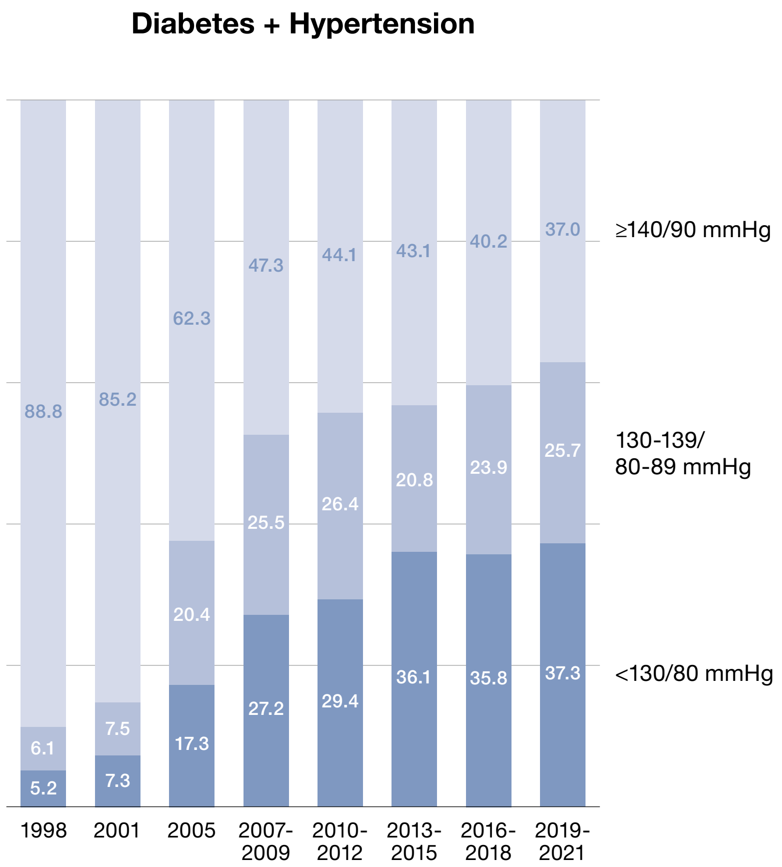

Diabetes defined as ① fasting glucose ≥126 mg/dL, ② physician diagnosis of diabetes, or ③ taking antidiabetic medication

Data Source: Korea National Health and Nutrition Examination Survey 1998-2021

Trends of Blood Pressure Control in People with Diabetes

Diabetes, Overall

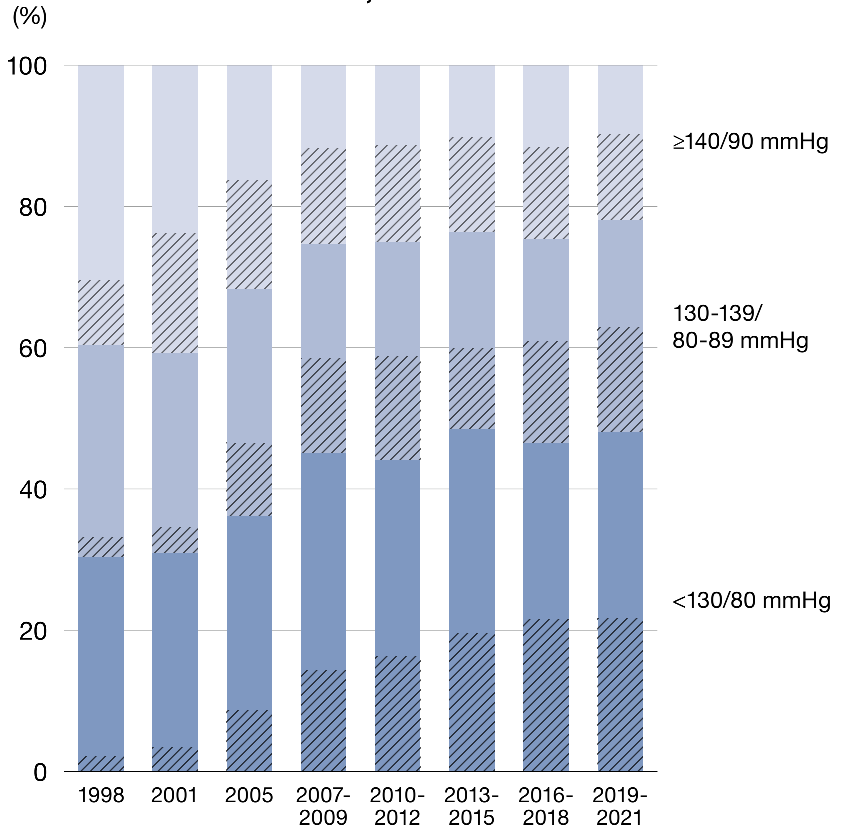

Diabetes + Treated for Hypertension

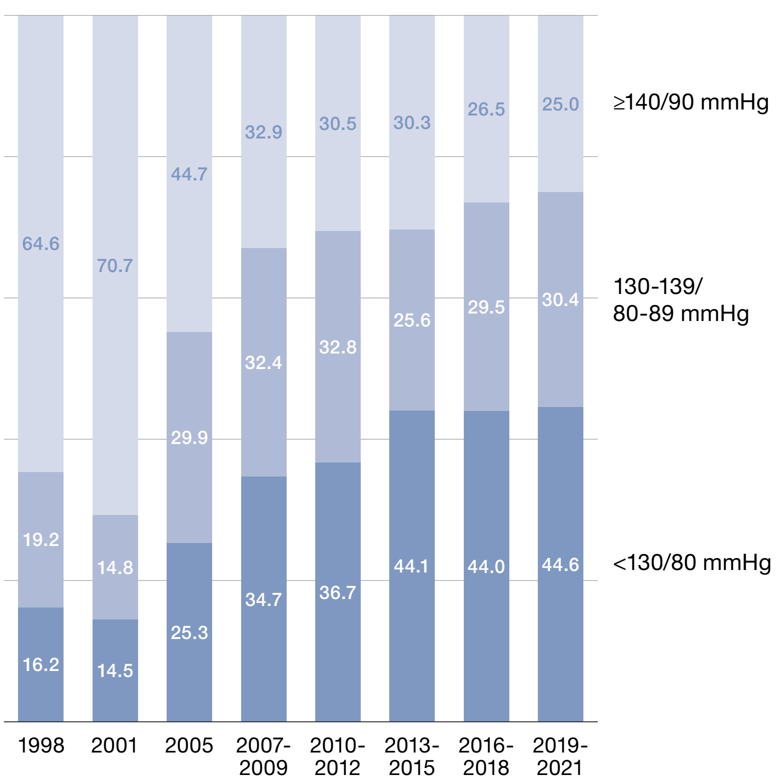

Diabetes defined as ① fasting glucose ≥126 mg/dL, ② physician diagnosis of diabetes, or ③ taking antidiabetic medication

Shaded area: People treated for hypertension

Data Source: Korea National Health and Nutrition Examination Survey 1998-2021

Trends of Blood Pressure Control in People with Obesity

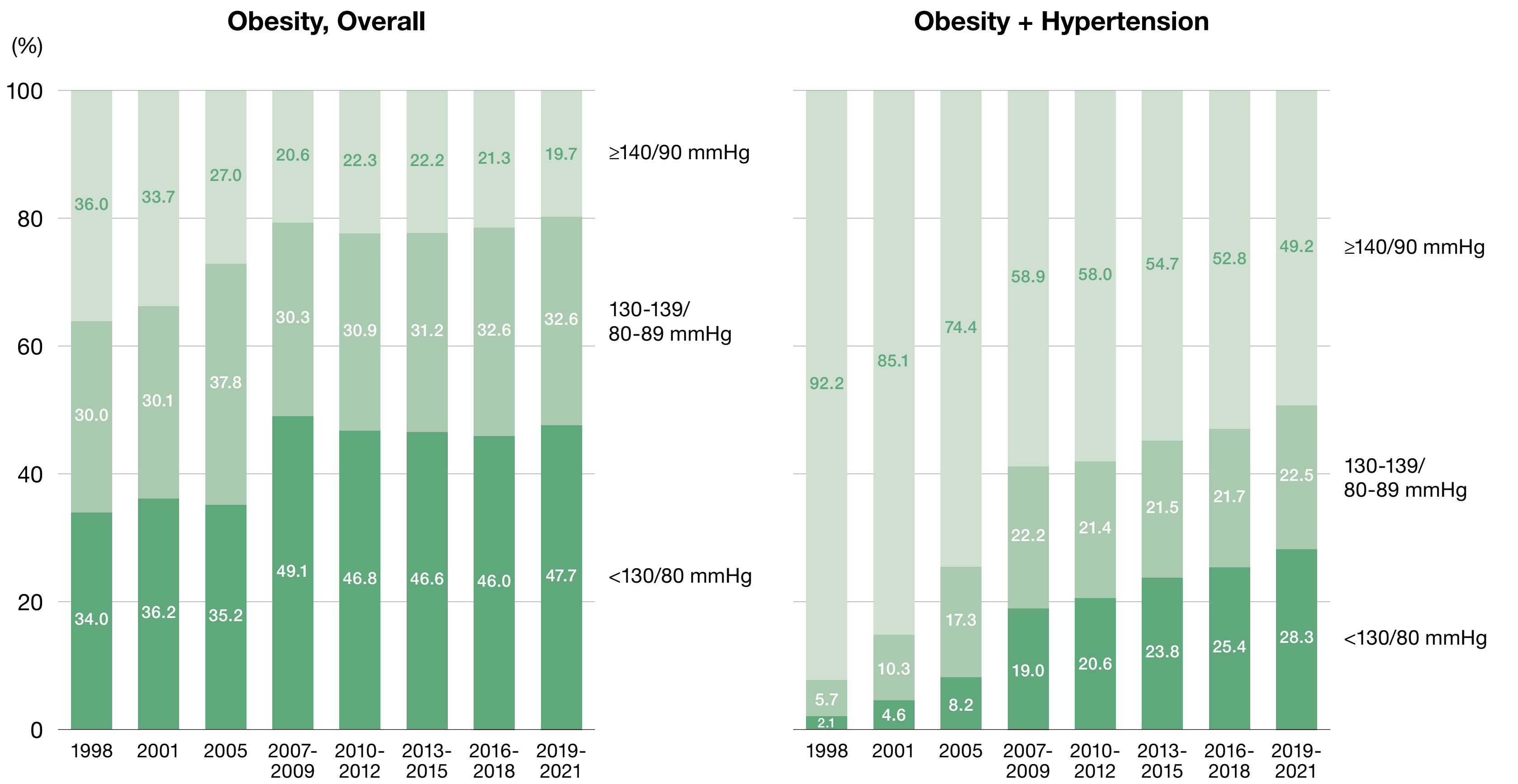

Obesity defined as body mass index ≥25 kg/m^2

Data Source: Korea National Health and Nutrition Examination Survey 1998-2021

Trends of Blood Pressure Control in People with Obesity

Obesity, Overall

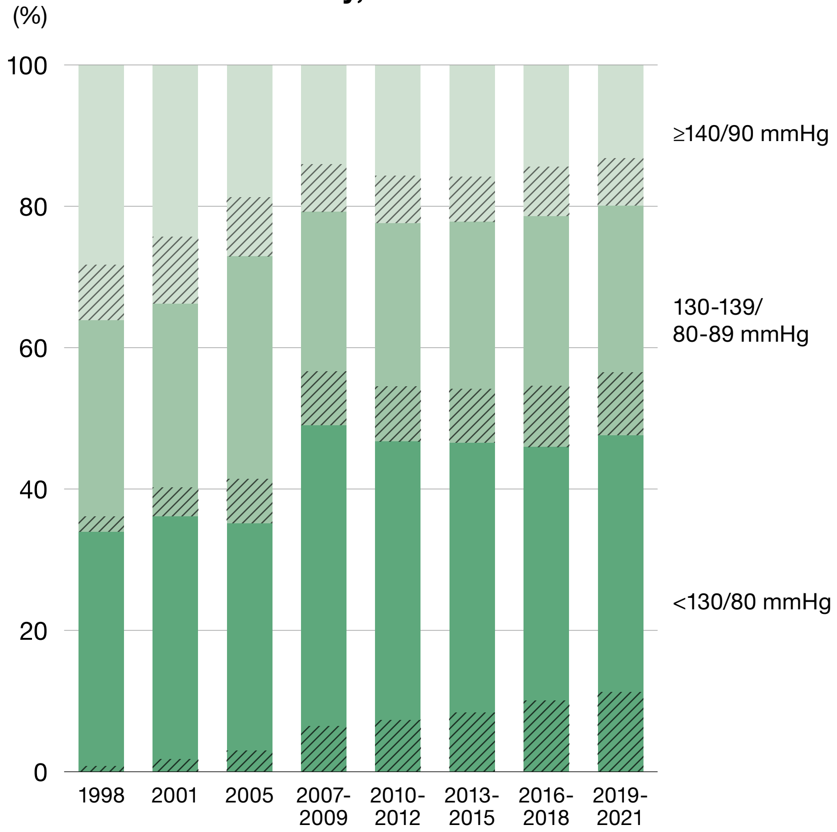

Obesity + Treated for Hypertension

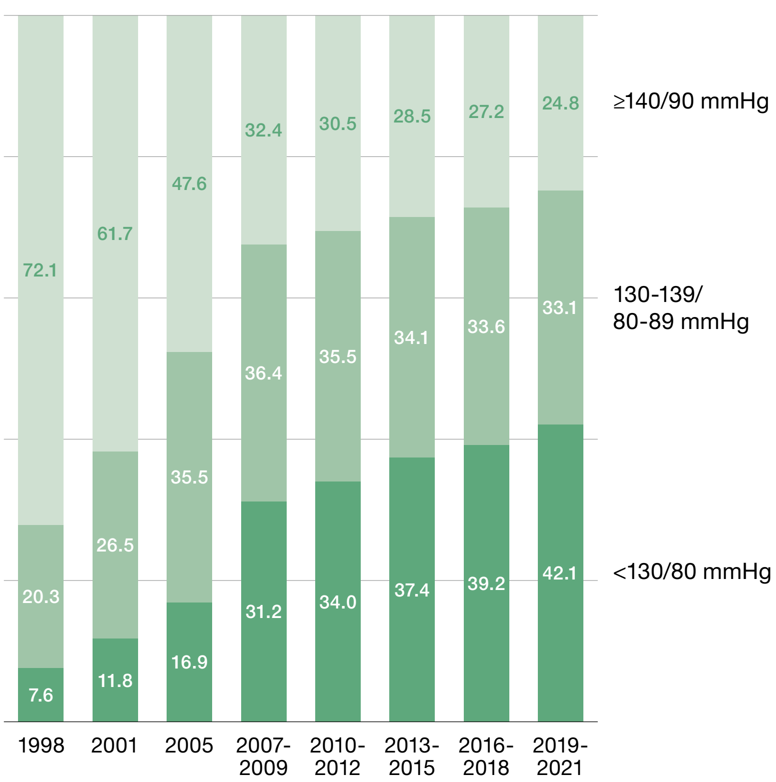

Obesity defined as body mass index ≥25 kg/m^2
Shaded area: People treated for hypertension

Data Source: Korea National Health and Nutrition Examination Survey 1998-2021

Trends of Blood Pressure Control in People with Chronic Kidney Disease

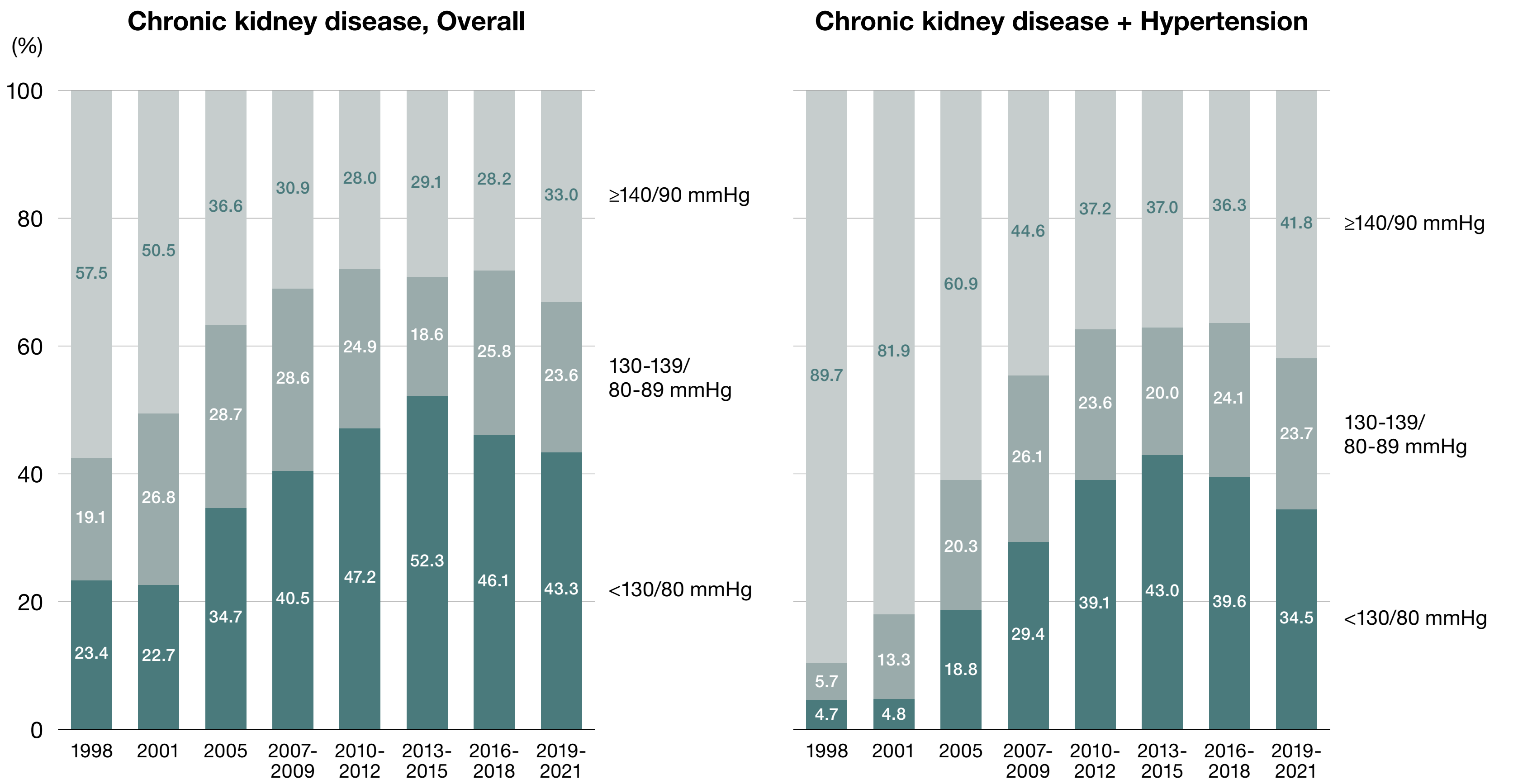

Chronic kidney disease defined as estimated glomerular filtration rate <60 mL/min/1.73 m^2 (calculated by CKD-EPI equation [2009])

Data Source: Korea National Health and Nutrition Examination Survey 1998-2021

Trends of Blood Pressure Control in People with Chronic Kidney Disease

Chronic kidney disease, Overall

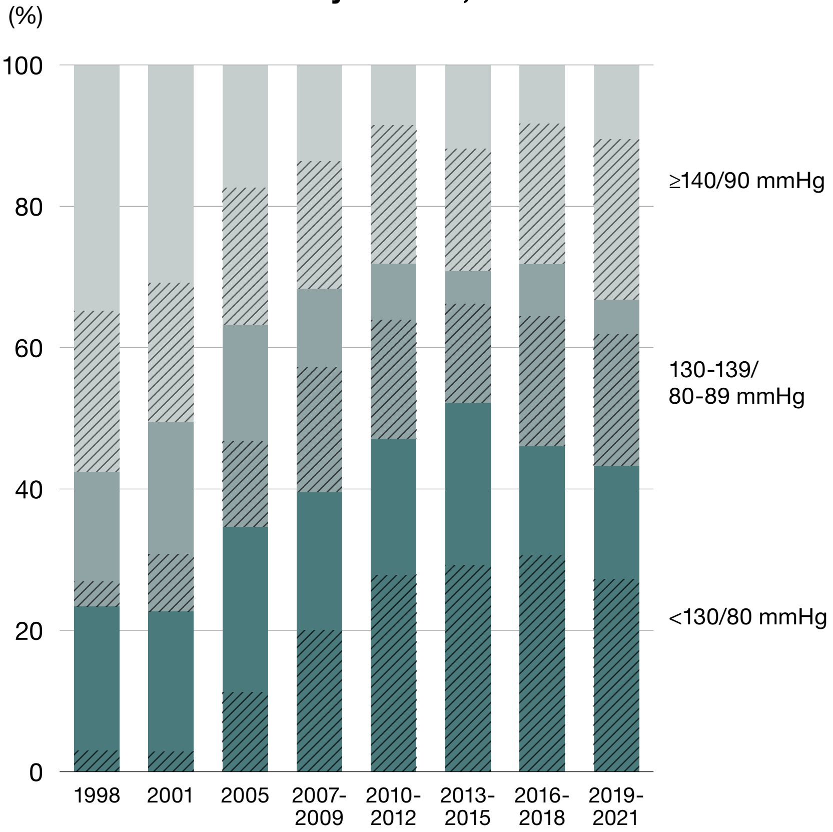

Chronic kidney disease + Treated for Hypertension

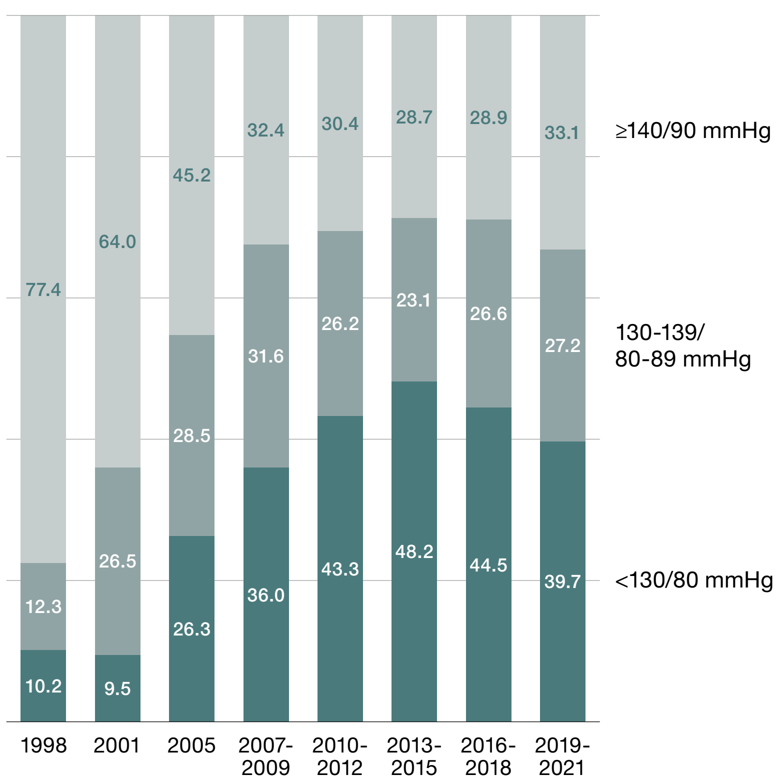

Chronic kidney disease defined as estimated glomerular filtration rate <60 mL/min/1.73 m^2 (calculated by CKD-EPI equation [2009])

Shaded area: People treated for hypertension

Data Source: Korea National Health and Nutrition Examination Survey 1998-2021

Trends of Blood Pressure Control in People with High-risk Hypertension

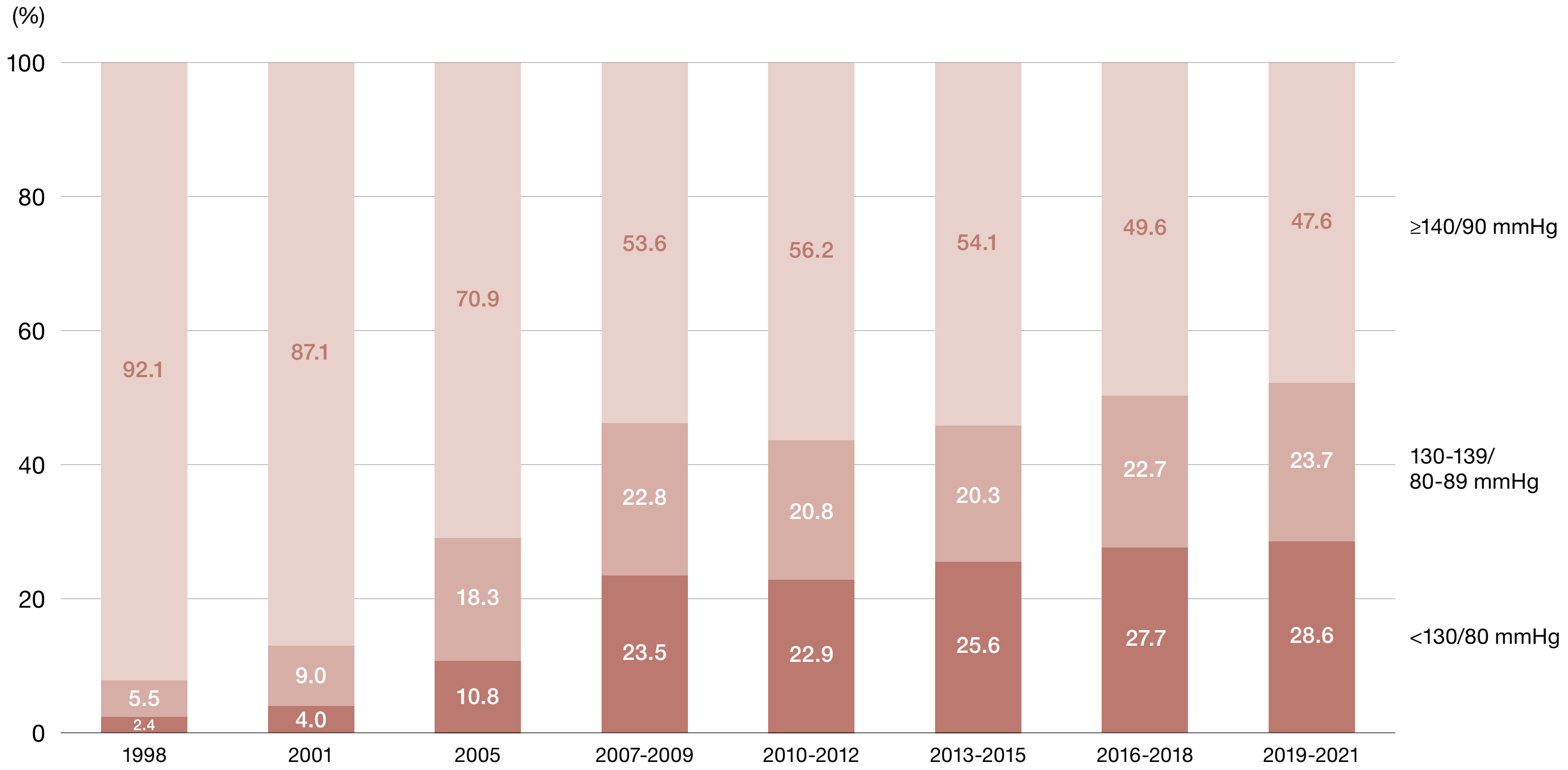

High-risk hypertension defined as hypertension with ≥3 of the following cardiovascular risk factors: old age (men ≥45, women ≥55; count as 2 if ≥65), family history of cardiovascular disease, current smoking, obesity or central obesity, dyslipidemia, prediabetes, and diabetes (count as 2).

· 1998, 2001: Information on antidyslipidemic medication use not available

· 1998, 2001, 2005, 2007-2009: Information on family history of cardiovascular disease not available

Data Source: Korea National Health and Nutrition Examination Survey 1998-2021

Trends of Blood Pressure Control in People Treated for High-risk Hypertension

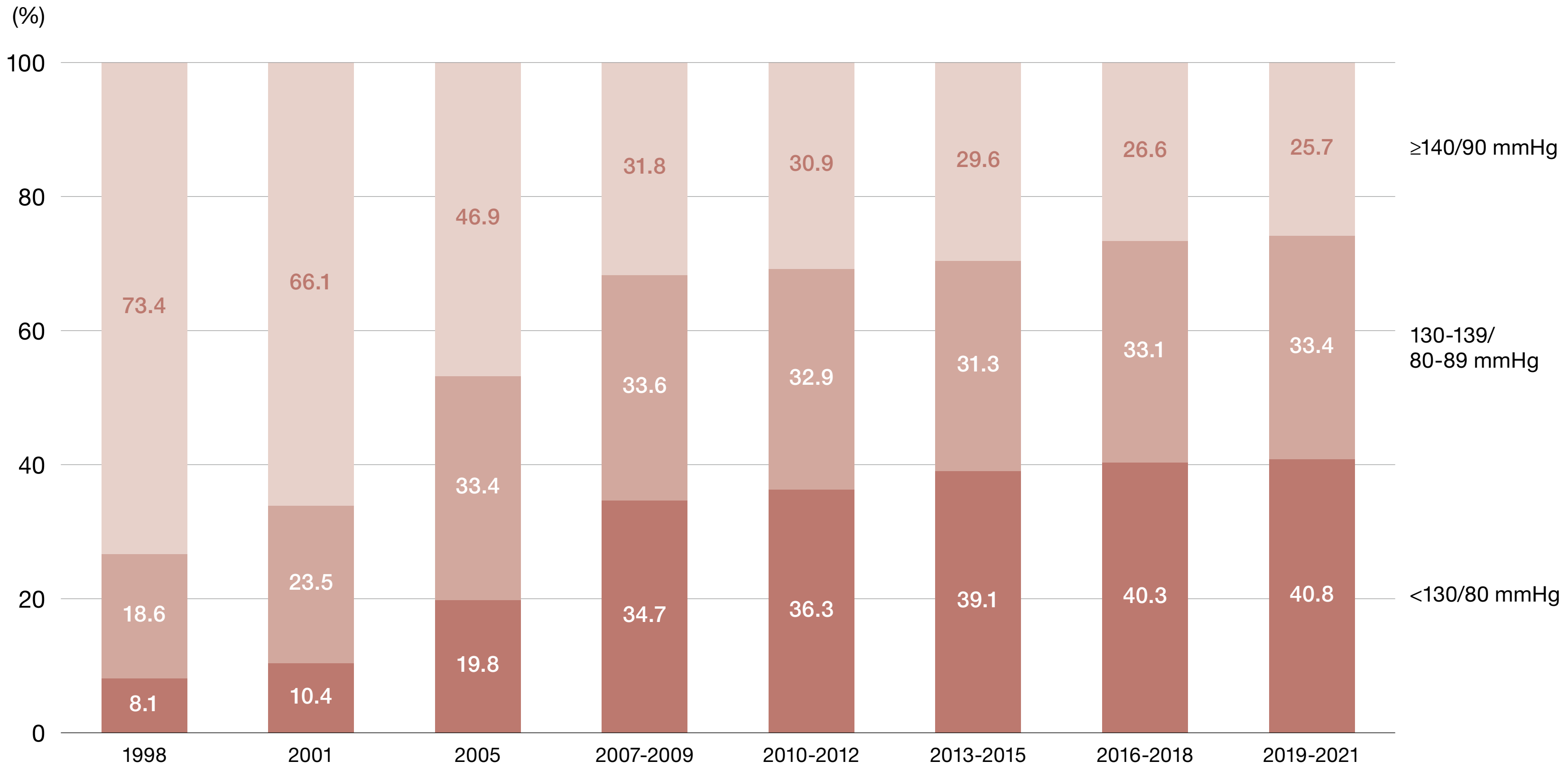

High-risk hypertension defined as hypertension with ≥3 of the following cardiovascular risk factors: old age (men ≥45, women ≥55; count as 2 if ≥65), family history of cardiovascular disease, current smoking, obesity or central obesity, dyslipidemia, prediabetes, and diabetes (count as 2).

· 1998, 2001: Information on antidyslipidemic medication use not available

· 1998, 2001, 2005, 2007-2009: Information on family history of cardiovascular disease not available

Data Source: Korea National Health and Nutrition Examination Survey 1998-2021

KOREA HYPERTENSION FACT SHEET 2023

This fact sheet will be published in Clinical Hypertension, the official journal of the Korean Society of Hypertension.
The Korean Society of Hypertension (KSH) - Hypertension Epidemiology Research Working Group.
Korea Hypertension Fact Sheet 2023. Clinical Hypertension 2023 (in press).

www.koreanhypertension.org
